# Using the DiCoT framework for integrated multimodal analysis in mixed-reality training environments

**DOI:** 10.3389/frai.2022.941825

**Published:** 2022-07-22

**Authors:** Caleb Vatral, Gautam Biswas, Clayton Cohn, Eduardo Davalos, Naveeduddin Mohammed

**Affiliations:** Open Ended Learning Environments, Department of Computer Science, Institute for Software Integrated Systems, Vanderbilt University, Nashville, TN, United States

**Keywords:** distributed cognition, learning analytics (LA), multimodal data, simulation based training (SBT), mixed reality (MR), DiCoT, human performance, multimodal learning analytics (MMLA)

## Abstract

Simulation-based training (SBT) programs are commonly employed by organizations to train individuals and teams for effective workplace cognitive and psychomotor skills in a broad range of applications. Distributed cognition has become a popular cognitive framework for the design and evaluation of these SBT environments, with structured methodologies such as *Distributed Cognition for Teamwork (DiCoT)* used for analysis. However, the analysis and evaluations generated by such distributed cognition frameworks require extensive domain-knowledge and manual coding and interpretation, and the analysis is primarily qualitative. In this work, we propose and develop the application of multimodal learning analysis techniques to SBT scenarios. Using these analysis methods, we can use the rich multimodal data collected in SBT environments to generate more automated interpretations of trainee performance that supplement and extend traditional DiCoT analysis. To demonstrate the use of these methods, we present a case study of nurses training in a mixed-reality manikin-based (MRMB) training environment. We show how the combined analysis of the video, speech, and eye-tracking data collected as the nurses train in the MRMB environment supports and enhances traditional qualitative DiCoT analysis. By applying such quantitative data-driven analysis methods, we can better analyze trainee activities online in SBT and MRMB environments. With continued development, these analysis methods could be used to provide targeted feedback to learners, a detailed review of training performance to the instructors, and data-driven evidence for improving the environment to simulation designers.

## 1. Introduction

Modern workplaces require workers to develop and execute a complex combination of cognitive, metacognitive, and psychomotor skills to achieve effective performance. With advanced technologies that have now become widely available, faster and more effective skill development can be achieved by designing effective training protocols that provide learners with multiple opportunities to train along with formative feedback to support continual improvement with clear pathways to achieve proficiency in their tasks. Simulation-based training (SBT) has become a popular paradigm to implement these training protocols. These environments provide safe and repeatable spaces for learners to practice and develop their workplace skills, and combined with adequate debrief and feedback they can support training in multiple domains (Ravert, 2002; Gegenfurtner et al., 2014).

When SBT scenarios require collaboration and feedback among multiple agents (real and virtual), it is common to interpret the training scenarios and trainee performance using theories of *distributed cognition* (Hollan et al., 2000; Hutchins, 2000). Furthermore, many SBT environments incorporate physical movement and embodiment, teamwork behaviors, and domain-specific tools to aid the workers, which match with the core tenets of distributed cognition (Kaplan et al., 2021). This is especially the case for SBT environments that are enhanced using mixed-reality tools, in domains such as emergency response, collaborative and embodied learning, and healthcare (Rosen et al., 2008; Mirchi et al., 2020; Rokhsaritalemi et al., 2020). Techniques such as *Distributed Cognition for Teamwork (DiCoT)* have been successfully applied to analyze SBT, both for the purposes of simulation design and learner feedback (Hazlehurst et al., 2008; Rybing et al., 2016, 2017). Traditionally, analysis of distributed cognition with these frameworks relies heavily on human observations by researchers and domain experts to provide a descriptive analysis of performance in the learning and training scenarios.

In parallel, other learning domains, such as K-12 classrooms, have seen a transformation in personalized learning through data-driven learner modeling and multimodal learning analytics (Hoppe, 2017; Ochoa et al., 2017). In these learning environments, data from student interactions are logged and analyzed to produce insights into the learners' cognitive, metacognitive, and affective processes, and the impact these processes have on their learning outcomes. While learning analytics has been employed to analyze learner performance in some simulation-based training domains as well, for example, in Biswas et al. (2019), Kim et al. (2018), and Martinez-Maldonado et al. (2020a), these applications are less common and often rely on cognitive theories derived from traditional learning frameworks. For learning and training in mixed reality-based simulation environments that involve multiple agents and combination of physical and virtual spaces, more advanced cognitive theories, such as distributed cognition, better match the affordances provided by the environments.

Motivated by this gap, in this paper we develop a framework to apply a *mixed quantitative + qualitative* approach that combines multimodal data analysis in the context of distributed cognition to analyze learner behavior and performance in SBT environments. In particular, our studies focus on a mixed-reality manikin-based (MRMB) environment for training nurses to work with patients in hospital rooms. MRMB-based simulation training provides realistic and high-fidelity scenarios for nurses to train in. They have proven to be quite effective in helping nurses develop and achieve proficiency in psychomotor, cognitive, and social skills as they interact with patients and equipment, make diagnoses, and provide interventions to alleviate their patient's problems (Hegland et al., 2017).

As a demonstration of our framework for tracking and analyzing trainee behaviors and performance, we ran a small study with nursing students in this MRMB training environment. We have developed and applied our mixed quantitative + qualitative methods approach to analyze the data collected with video, audio, and eye tracking sensors. Our computational architecture processes the raw multimodal data streams and analyzes this data framed using the constraints and insights derived from a qualitative analysis using the DiCoT distributed cognition approach. The results are mapped to a combined qualitative-quantitative representation of the nurses' problem solving behaviors and performance, with the help of our cognitive task model. With continued development and refinement, results from our analysis methods can be provided to learners as formative feedback and to instructors to help them guide more detailed discussions during simulation debriefing.

The analysis presented in this paper supports an investigation of two primary research questions:

1. How can multimodal learning analysis be used to support a comprehensive analysis of distributed cognition in MRMB simulation training environments?
2. How does temporal alignment and analysis of multiple data modalities help us gain a deeper understanding of trainees' actions in the context of the tasks they are performing in an MRMB environment?

The rest of this paper is organized as follows. Section 2 presents previous work on SBT, the Distributed Cognition framework, and an overview of multimodal data analysis approaches applied to studying learner behaviors. Section 3 discusses our theoretical framing of the training scenarios and analysis by combining cognitive task modeling, distributed cognition through the DiCoT methodology, and multimodal data analytics. Section 4 provides details of the methods we have adopted in our study; first an overview of the MRMB-based Nurse Training scenario, a Cognitive Task Analysis approach to interpreting and analyzing nurses' actions in the training environment and mapping them to higher level cognitive behaviors, our adaptation of the DiCoT framework to study nurse performance and behaviors in the training scenarios, and a complete computational architecture to derive performance analysis from data collected in the SBT environment. Section 5 presents details of the analyzes of the nurses' performance and behaviors in the case-study MRMB-based training environment. This is followed by a discussion of the results obtained for two the scenarios and their broader implications in Section 6. Last, Section 7 provides the conclusions of the paper, limitations with the current approach, and directions for future work.

## 2. Background and related work

In this section, we briefly review past work in SBT, distributed cognition, and multimodal analytics applied to analyzing learners' training performance and behaviors.

### 2.1. Simulation-based training

Simulation-based environments offer many attractive properties for training applications; they provide controllable and repeatable environments in which learners and trainees can safely practice complex cognitive and psychomotor skills in rich and dynamic scenario representations. Thus, it is not surprising that simulation-based training has been widely adopted for a variety of domains, and many studies have shown them to be effective for both training and assessment (Ravert, 2002; Maran and Glavin, 2003; Daniels and Auguste, 2013; Rybing, 2018). In medical domains, SBT has been used since the 1950s when the first commercial medical training manikin was released. The manikin-based approach combined with computer-based simulations continues to be widely utilized and studied today (Cooper and Taqueti, 2008; Hazlehurst et al., 2008; Pimmer et al., 2013; Rybing et al., 2017). For example, Rybing et al. (2017) studied the use of simulation-based training for nurses in mass causality events; Kunst et al. (2016) studied the use of manikin simulation for mental health nursing; and Johnson et al. (2014) found that mankin-based education was more effective than web-based education for advanced practice nursing students. For further information, see Cooper and Taqueti (2008) which reviewed the history and development of manikin-based clinical education, Al-Ghareeb and Cooper (2016) which reviews the current state of manikin-based clinical education along with its barriers and enablers, and Gegenfurtner et al. (2014) which reviewed the larger context of digital simulation-based training.

In addition, the integration of simulation environments with advanced computing resources has led to further advances in the field. Computer-based simulations allow for automated collection of trainee activity data, which can then be used to evaluate their performance, and for debriefing and after-action reviews (Ravert, 2002; Sawyer and Deering, 2013). In medical domains, a lot of the computer-based simulation training relies on high fidelity manikins that trainees can realistically interact with to practice their clinical and teamwork skills (Al-Ghareeb and Cooper, 2016). This creates *mixed-reality* environments, where trainees act in a physical space, which includes real equipment that interfaces with a digital simulation. The digital simulation controls the patient manikin's vital signs and overall health manifestations. In addition, the digital simulation can take into account trainees actions in the environment and on the manikin, and adapt the manikin's vital signs and responses to these actions.

The overall goal of SBT is to help learners to develop a set of skills that are *transferable*, meaning the skills acquired in the simulation can be utilized in other simulation settings and in real-world situations. In particular, one of the primary goals for medical SBT is to help trainees develop skills that transfer from the simulation environments to actual medical settings with real patients. *Application validity* measures capture how well SBT environments accomplish this transfer for a sufficiently large population of trainees (Feinstein and Cannon, 2002).

Prior work has shown that providing formative feedback during debrief after the simulation improves both the application validity of the simulation, as well as the competence and self-efficacy of the learners (Gegenfurtner et al., 2014). It is important to note that the formative feedback provided must be discussion and explanation focused, and not purely evaluative in order to preserve the psychological safety of the training environment (Kang and Min, 2019). While similar simulation environments are also used for learner assessment (Cook et al., 2014), our focus in this paper is on simulation-based *training*, where learners must feel safe to practice and not fear that mistakes will have long-term negative consequences (Kang and Min, 2019; Park and Kim, 2021). Taking this into account, our work focuses on building analysis methods designed to provide feedback that will guide and support discussion and learning during debrief. Our analysis methods are based on multimodal data generated by the mixed-reality environment grounded in the theory and practice of distributed cognition.

### 2.2. Distributed cognition

Traditionally, cognition is studied with the individual as the basic unit of analysis. In essence, this classical view of cognition views the brain of an individual as a processing unit, which takes input from the outside world, manipulates this information, and produces some output, often in the form of body functions, such as movement and speech (Clark, 1997). However, this view of an individual mind as the basic unit of cognition ignores the complex relationship between the mind, the body, and the larger environment. The ability to leverage movement, tools, technology, collective wisdom, and social structures allows humans to achieve far more than an isolated individual mind alone can, but the traditional view of cognition marginalizes these embodied, cultural, and environmental components (Geertz, 1973; Hazlehurst et al., 2008).

These limitations with classical cognition led some cognitive scientists, such as Clark, Hutchins, Cole, and others in the late twentieth century to begin developing alternative systems of examining cognition (Hutchins, 1995; Clark, 1997; Cole, 1998). One such alternative approach is *Distributed Cognition*, developed by Hutchins and colleagues (Hutchins, 1991, 1995, 2000, 2006). Distributed cognition (DCog) extends the boundaries of classical cognition from the mind of an individual in isolation into a collective that includes the individual's mind, body, other people, and the environment in which the cognition is taking place. Instead of the unit of cognitive analysis being the individual mind, distributed cognition treats the entire activity system as the unit of analysis, with the goal of understanding cognition at this system level (Hazlehurst et al., 2008; Rybing, 2018).

Hutchins argues that cognition occurs across at least three different modalities (Hutchins, 2000). First, cognition can be distributed across members of a *social group*. This can be seen as individuals coming together to solve a problem and contribute to a common goal. Second, cognition can be distributed between *internal* and *external structures*. This is most evident in the use of tools, where individuals offload some cognitive processing to a material or environmental object, but can also have some less apparent manifestations, such as the layout of a physical space affecting cognition. Third, cognition can be distributed across *time*, with the nature and outcomes of earlier events affecting the nature of later events (Hutchins, 2000).

Distributed cognition is particularly relevant in analyzing training performance and behaviors in mixed-reality, simulation-based training. Mixed-reality SBT environments manifest many of the characteristics of these three distributed modalities. SBT inherently contains social structures and roles over which cognition is distributed. When multiple learners train simultaneously in the environment, the social distribution and interactions can be studied explicitly, with the learners collaborating and sharing the cognitive load and decision making processes in the task. Even in SBT cases with only one learner, there is a social distribution between the learner and the instructor, with information traveling and transforming between the instructor and student as they interact. SBT also contains instances of cognition distributed between internal and external structures. In mixed-reality scenarios, there is a distribution between the learners' minds, the physical space they inhabit, and the digital space with accompanying interfaces that are controlled by the simulation. In addition, many training domains require learners to learn and operate domain-specific tools, which also represent artifacts of distributed cognition. Finally, SBT is necessarily temporal, as learners practice skills that change (improve or degrade) over time. Thus, previous practice and previous actions will affect the ways in which learners approach current cognitive tasks.

Other studies which focus on nursing simulation-based training have also adopted distributed cognition for their analysis. Rybing et al. (2017) use distributed cognition to analyze nursing students training on a mass causality simulation; Pimmer et al. (2013) contrast various cognitive theories used in clinical learning to highlight advantages of distributed cognition; and (Hazlehurst et al., 2008) discuss the use of distributed cognition as a framework for medical informatics. Because of this overlap between the distributed cognition framework and the modeling and interpretation of learner behavior in simulation based training in general, and in medical and nurse training in particular, we ground our analysis methods using Distributed Cognition as a theoretical framework.

### 2.3. The DiCoT analysis framework

Despite the advantages of distributed cognition as a cognitive framework, application of the framework requires specific methodologies that are not outlined in the original work. Several structured qualitative analysis methodologies have been developed for analyzing distributed cognition in different domains and scenarios. For example, Wright et al. (2000) proposed the *Resource Model* to study human computer interaction in a team framework, Galliers et al. (2007) proposed the *Determining Information Flow Breakdown (DIB)* model to study organizational learning in response to adverse events in medical settings, and Stanton (2014) proposed the *Event Analysis of Systemic Teamwork (EAST)* framework that employs three network models (i.e., task, social and information) to analyze the interactions between the sound room and control room in a submarine. Following the wide adoption of distributed cognition models and their success in analyzing trainee behaviors in the medical training domain (e.g., Hazlehurst et al., 2008; Pimmer et al., 2013; Rybing et al., 2017), we adopt the *Distributed Cognition for Teamwork (DiCoT)* model proposed by Blandford and Furniss (2006).

**Figure 1:**
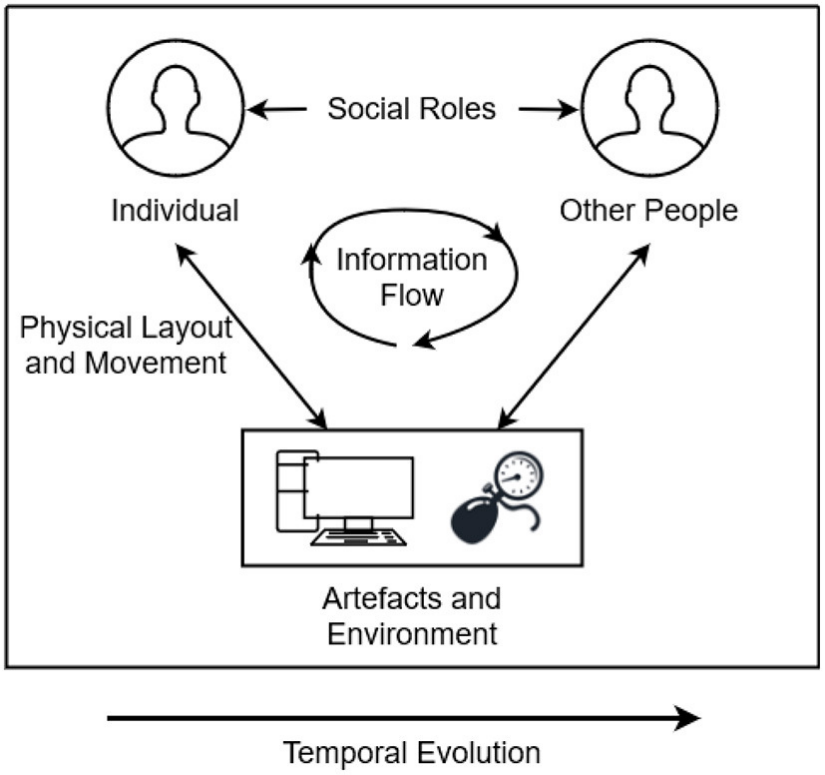
Illustration of the interactions between each of the five DiCoT themes and how they work together to construct the entire cognitive system.

DiCoT is a qualitative analysis framework designed to analyze distributed cognition by breaking down a cognitive system into five independent themes: (1) *information flow*, (2) *artifact and environment*, (3) *physical layout*, (4) *social interactions*, and (5) *temporal evolution* (Blandford and Furniss, 2006). The information flow model focuses on how information propagates and transforms through the system. The artifact theme follows how tools can be used to aid the cognition of the system. The physical layout theme examines the way in which objects and people are arranged in a space and how that arrangement affects cognition. The social model focuses on the relationships between people in the cognitive system and the individual's differing knowledge, skills, and abilities. Finally, the temporal evolution model focuses on how the system changes over time. Each of the five DiCoT themes contributed components to our understanding the overall cognitive processes and psychomotor skills that trainees employed in the environment, but the themes are also highly interconnected. Figure 1 illustrates how the themes manifest within a cognitive system and the various ways in which the different themes interact with one another. For example, social roles mediate how information flows between different individuals; physical layout mediates how information flows between individuals and the environment; and temporal evolution describes and mediates how these processes change over time. By analyzing each of the themes individually and how each theme interacts with the others, we can fully understand the distributed cognition processes and psychomotor skills being enacted in the system.

In order to analyze each of the themes and their interactions, the DiCoT methodology defines several *principles* that describe the ways in which each component of the system contributes to the overall cognitive process. For example, principle 10: Information Hubs, describes that certain artifacts in the system are central focuses where different channels of information meet. This principle is primarily related to the *information flow* and *artifacts and environment* themes. By analyzing the different artifacts in a distributed cognitive system and how they are referenced for information, we can determine which artifacts represent information hubs and how the design of those hubs influences the overall cognition of the system. Each of the 18 principles is analyzed in a similar way, but relate to other components of the system. All eighteen DiCoT principles are summarized in Table 1. By analyzing the distributed cognition system and identifying the manifestations of each of these 18 principles within the system, we can understand how each of the 5 DiCoT themes work together to construct the overall cognition of the system. We discuss our qualitative analysis of the nurse training simulation using DiCoT framework in Section 4.3.

**Table 1:** The 18 principles of DiCoT analysis, summarized from Blandford and Furniss (2006).

**Principle name**	**Description**
1. Space and cognition	The role space and spatial layout play in supporting cognition
2. Perceptual principle	Spatial representations support cognition more than non-spatial representations, as long as there is a clear mapping between the space and that which the space represents
3. Naturalness principle	Cognition is aided when the form of a representation matches the properties of what it represents
4. Subtle bodily supports	Individuals often use their body to support cognitive processes
5. Situation awareness	People need to be informed of and understand what has previously happened, what is currently going on, and what is planned
6. Horizon of observation	The information that can be seen or heard by a person; closely related to and influencing situation awareness
7. Arrangement of equipment	The layout of equipment affects what information people have access to, and thus their ability to process it
8. Information movement	Information moves around a system in a number of ways, which all have unique functional consequences
9. Information transformation	Information can be represented in many forms, and often must transform between these forms when moving and when being processed
10. Information hubs	A central focus or source where different channels of information meet and are processed together
11. Buffering	If incoming information interferes with ongoing activities, buffering allows the information to be held until an appropriate time where it will not interfere
12. Communication bandwidth	Different modalities of communication often carry different amounts of information. For example, face-to-face communication offers more information than computer-mediated communication
13. Informal communication	Not all communication is formal, and sometimes informal communication can carry very important information that is not otherwise passed
14. Behavioral trigger factors	Groups of people can operate together without an overall plan by individually responding appropriately to certain local trigger factors
15. Mediating artifacts	People often bring artifacts into coordination to support completion of a task
16. Creating scaffolding	People often simplify their cognitive tasks by utilizing their environment
17. Representation-Goal Parity	When an artifact is used to represent the system's goal, representations closer to the goal of the user are more powerful
18. Coordination of Resources	Different information structures can be coordinated to aid in cognition

### 2.4. Multimodal learning analysis

Learner modeling based on student performance and behavior has been the cornerstone for adapting and personalizing computer-based learning environments to individual learner needs. More recently, data-driven approaches to learner modeling based on learning analytics and machine learning methods have become popular for capturing and analyzing learner behaviors in complex instructional and training domains (Hoppe, 2017). With the development of these data-driven learning analytics techniques gives rise to the question: *What forms of data need to be collected to enable meaningful analysis in specific learning scenarios*? In traditional computer based learning environments, typical data collection includes interactions with the system that can be logged. Analysis of the logged data paints a reasonable picture of the learners' activities in the context of the tasks they are performing in the environment (Hoppe, 2017; Ochoa et al., 2017).

However, more recent work has begun to point out the potential limitations of these traditional methods. By only using logged data that is easy to collect, we may miss out on important context and interpretation that the information sources may provide. Therefore, we may require additional sensors to collect such data (Ochoa et al., 2017). In other words, to aviod the so-called *streetlight effect* (Freedman, 2010), researchers have begun to consider alternative and more complex data sources, such as physical movement, gestures, and posture captured with video; dialogue captured using microphones; stress levels captured with biometric sensors; and gaze and attention collected using eye tracking devices. Data collected using these modalities become especially important when the learning or training task requires operations in physical or mixed-reality spaces, and when learners work in groups to accomplish overall goals.

Combining all of the modalities of operation (e.g., activities, communication, affective states, stress levels, and gaze) can lead to analyzes that provide a more complete picture of the cognitive, psychomotor, and metacognitive processes of the learners (Blikstein and Worsley, 2016). The focus on collection, processing, and analysis of this quantity and variety of data has been the basis for new research and analyzes in the field of multimodal learning analytics (MMLA) (Blikstein, 2013; Blikstein and Worsley, 2016; Worsley and Martinez-Maldonado, 2018). These new MMLA methods have also been applied to simulation-based training environments. For example, Martinez-Maldonado et al. (2020b) examined how to design actionable learning analytics for manikin-based nurse training; Di Mitri et al. (2019) designed MMLA methods for detecting mistakes during CPR training; and López et al. (2021) studied collaborative behaviors in serious tabletop games using MMLA methods.

In our own previous work, we have applied MMLA methods to analyze teamwork behaviors in simulation-based training environments, including those that incorporate mixed-reality components (Biswas et al., 2019; Vatral et al., 2021, 2022). Our analyzes of learner performance and behaviors have been based on *cognitive task analysis*, which is a set of methods commonly used to describe and decompose complex problem-solving domains into their core cognitive proficiencies (Clark and Estes, 1996; Schraagen et al., 2000; Zachary et al., 2000). These cognitive components describe multiple parameters that include goal setting, planning, decision making, declarative and procedure knowledge and execution, and situational awareness (Militello and Hutton, 1998). The models and insights generated from the task analysis are often critical in the design and development of training systems for these complex domains.

### 2.5. Cognitive task analysis

Cognitive Task Analysis typically draws from multiple sources. This includes a review of relevant literature, interviews with domain experts, and observing and interpreting training activities in the mixed reality simulation environment in terms of their conceptual and procedural components. From this analysis, one can build a comprehensive task-subtask hierarchy that links high-level tasks and subtasks down to specific observable skills and activities performed by trainees (Biswas et al., 2019; Vatral et al., 2021). The hierarchy is designed to support the inference of complex cognitive concepts by analyzing observable behaviors and data. Cognitive processes related to task execution are located at the highest level of the task hierarchy, and each deepening level representing more concrete and observable manifestations of these concepts within the task domain.

By analyzing the observable multimodal data at the lowest levels of the hierarchy and propagating the results up to higher levels, we can generate inferences about cognitive activities and competencies of trainees. In this way, insights generated from cognitive task analysis combine top-down model-driven and bottom-up data-driven approaches. In previous work, we have applied cognitive task analysis methods to demonstrate how teamwork in mixed-reality SBTs can be evaluated using MMLA (Vatral et al., 2022). In this paper, we extend this work and further ground the MMLA analyzes methods in distributed cognition, as described in the next section.

## 3. Theoretical framework

Our goal in this work is to present a framework for combining the benefits and insights from qualitative analysis of distributed cognition through the DiCoT methodology and quantitative analysis through data-driven multimodal analytics. Analysis using qualitative methods (Cognitive Task Analysis, DiCoT) provides domain semantics to inform how the quantitative analysis (MMLA) is performed, and in turn, results of the quantitative analysis provide new insights into the domain and the learner behaviors that inform a richer qualitative analysis. We believe that by presenting an integrated qualitative and quantitative analysis that inform and shape one another, the strengths of each method can be amplified, thus providing for a deeper insights than each method individually and better feedback to learners, instructors, simulation designers, and researchers.

**Figure 2:**
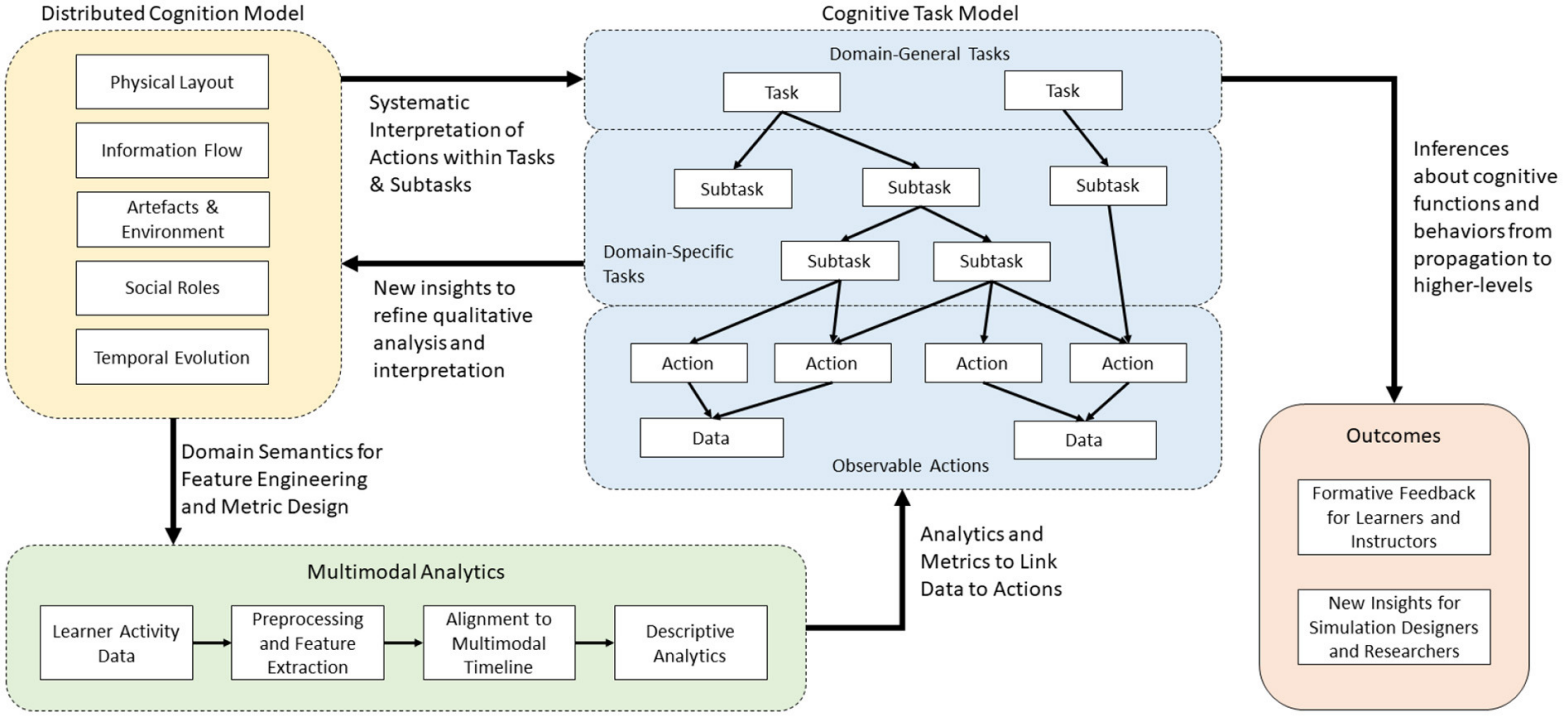
The overall theoretical framework to combine qualitative DiCoT analysis with quantitative multimodal analytics for understanding learner behaviors in simulation-based training.

Our overall theoretical framework, illustrated in Figure 2, breaks down this cyclic combined qualitative and quantitative analysis approach into three major components: DiCoT analysis, multimodal analytics, and the cognitive task model. The cognitive task model provides the cornerstone of the overall framework. For our MRMB simulation environment, we frame the task model around the set of primary tasks that define the training or learning domain. These concepts represent the mapping of the task domain into the overarching cognitive processes, psychomotor skills, affective states, and collaborative processes that are relevant to the task domain. For example, learning and training domains typically include high level cognitive processes such as information acquisition, problem solving, solution construction, solution testing, and evaluation. These processes, in a broad sense, remain invariant across multiple domains and training scenarios. However, their interpretation and execution may differ depending on the training scenario and the domain under consideration.

Next, we construct the hierarchical structure by breaking down the highest level cognitive, psychomotor, affective, and collaboration concepts into their more domain specific sub-components and sub-tasks using a progressive elaboration process. The primary reason for creating the different levels of abstraction is to ensure that variations of training scenarios, though they may differ in their lower-level task definitions and sub-divisions, map onto relevant higher level processes and help define proficiency measures in the task domain.

In more detail, primary tasks are decomposed into sub-tasks; sub-tasks are further decomposed into more fine-grained sub-tasks; and so on until we reach a set of basic task units that cannot be meaningfully decomposed further. We call this basic unit an *action*. Each sub-task represents a constituent requirement that is sequenced and completed to accomplish the higher-level tasks in the layer above them. Moving toward the lower levels of the hierarchy, the sub-tasks become more and more domain-specific, and at the lowest levels map onto observable actions and behaviors. For example, consider information-acquisition as the highest-level cognitive task. In order to acquire information, we may visit a library, search the internet, ask a friend, and so on. The specific sub-tasks included within the task model are limited by the domain being analyzed. By limiting each level to sub-tasks specific to the given domain, we follow a top-down approach to modeling and produce a task space model of the domain.

While the modeling of the domain is approached top-down, the interpretation of the learner actions and behaviors uses the model in a bottom-up manner, interpreting the multimodal data collected from the environment into lower level activities and behaviors. We employ a variety of multimodal analysis techniques to link from observable data back to the interpreted actions performed by the learners. This is illustrated by the arrow linking multimodal analytics (green) to the cognitive task model (blue) in Figure 2. The specific analytics and algorithmic methods utilized depend on the domain being analyzed and the specific sensors that are available. For example, if microphones are available, we can apply natural language processing algorithms to convert the audio signals to semantic information on the topic of the conversation. Similarly, if we collect video data, then computer vision techniques can be used to understand movement actions within the simulation space. The design of these analytics and algorithmic methods within a specific domain are informed by the qualitative DiCoT analysis, as illustrated by the arrow linking distributed cognition (yellow) to multimodal analytics (green) in Figure 2. By analyzing the training environment using the DiCoT methodology, we can determine important components of the task domain that inform the categories and classes for the algorithmic models.

For example, in our nursing domain, the DiCoT analysis revealed that there are four meaningful semantic areas in the simulation space: left of the bed, right of the bed, foot of the bed, and outside the room (see Section 4.3.1). Thus, we can adopt this result from the qualitative analysis into the design of the quantitative algorithmic methods by using the video data to determine when the nurses move between each of these four semantic regions (see Section 4.4.1). As a second example, in our nursing domain, the DiCoT analysis revealed the various artifacts that are semantically important to information flow (see Section 4.3.2). We can adopt this result by using the eye-tracking gaze data and mapping the raw x-y gaze position data onto instances where the nurse is looking at each of the semantically important artifacts identified by the DiCoT analysis (see Section 4.4.4). In this way, we use the results of DiCoT analysis to create algorithmic models that convert raw data (e.g., video, audio, etc.) into action- and behavior-level interpretations.

Once we convert from the raw data to the action- and behavior-level interpretations, they are mapped onto a common *timeline*. As a next step, we can develop algorithms to interpret temporal sequences of actions and behaviors, and roll them up into upper sub-task levels. Some actions only contribute to a single sub-task, but others may link to multiple sub-tasks. These multiple hierarchical links in the task model add expressivity to our task models, but may make the analysis process more challenging because multiple inferences may have to be made on similar action sequences using additional context information.

We systematize this interpretation process by once again introducing results from the qualitative DiCoT analysis of the task environment, as illustrated by the arrow linking distributed cognition (yellow) to the cognitive task model (blue) in Figure 2. Results from the DiCoT analysis can provide semantic context to the interpretation of learner actions within the environment, and map them onto the sub-tasks to which the individual action may contribute. For example, when analyzing a group of participants in a restaurant, collected sensor data, such as video analysis or accelerometers, may indicate that a specific participant was performing the action of cutting with a knife. This action may contribute to at least two potential disjoint sub-tasks of interest: eating food or cooking food. However, based on a previous DiCoT analysis of the environment, we know that the physical layout of the restaurant strongly mediates the interpretation of these two sub-tasks; cooking activities occur in the kitchen, while eating activities occur primarily in the dining room. By adding this semantic context derived from the physical layout theme of the DiCoT analysis, we know that we can simply look at the participant's position in the restaurant to disambiguate this knife cutting action. As an extension, if we captured participant dialog and additional video around the cutting event, we may use information flow DiCoT theme to analyze the motivations for this action within a given sub-task, for example, to deduce that one participant was dividing his portion of food to share with another as part of the eating process.

While this restaurant scenario analysis represents a simplistic example, it demonstrates the second way in which DiCoT is important for adding semantic context to our computational analysis. First, DiCoT informs the design of algorithms and models to convert raw data to action-level interpretations. Second, DiCoT provides context-specific disambiguation when mapping lower-level action and sub-tasks onto high-level tasks and sub-tasks. By iterative analysis, we can propagate learners' activities up to the highest-levels of the task model to understand their cognitive, psychomotor, affective, and collaborative behaviors.

By presenting the learners and instructors with quantitative metrics and qualitative descriptions of learner activities at multiple levels of the task model hierarchy, we can provide a basis for further discussion at different levels of detail during simulation debrief, while also tracking progress and changes in learner behavior over time. In addition, the results generated from this computational analysis also provide additional insights back into semantic models of the domain and inform a richer qualitative (DiCoT) analysis and task model construction. This idea is illustrated by the cyclic link from the cognitive task model (blue) to distributed cognition (yellow) in Figure 2. For example, analysis of the data might reveal certain learner behaviors that are not well accounted for in the current DiCoT analysis and task models. By presenting this result back to researchers, these analysis models can be refined and updated to contain a more complete understanding of the task environment and learner behaviors. This creates the loop back in our framework. Qualitative DiCoT and task analysis methods provide domain semantics and systematic methods for interpreting collected learner data, and the analysis of collected learner data reveals new insights that can be used to refine the DiCoT and task models. In the next section, we apply our task modeling framework combined with the DiCoT and multimodal analyzes to our MRMB nurse training case-study.

## 4. Methods

In this section, we demonstrate application of our theoretical framework to a small case study of nurses training in an MRMB environment. We begin with a complete description of the case study, including description of the affordances of the simulation environment and all of the data that was collected for the analysis. After this, we show how each of the three components of our theoretical framework apply to interpreting and analyzing nurses' activities and behaviors in this domain. First, we explain the construction and structure of the complete cognitive task model, from the high-level abstract cognitive tasks down to specific actions and observable data. Second, we describe a DiCoT analysis of the training environment, explaining each of the five themes in depth. Finally, we present a computational architecture, based on multimodal analysis, which tracks the raw multimodal data collected from the training environment through the cognitive task model to generate inferences, analytics, and performance metrics that describe the nurses' training behaviors within the context of the distributed cognition system.

Following the description of each component of the theoretical framework applied to the case study, we demonstrate the processes of following the collected data through the framework to generate inferences about nurse behaviors.

### 4.1. Case study-MRMB nurse training

**Figure 3:**
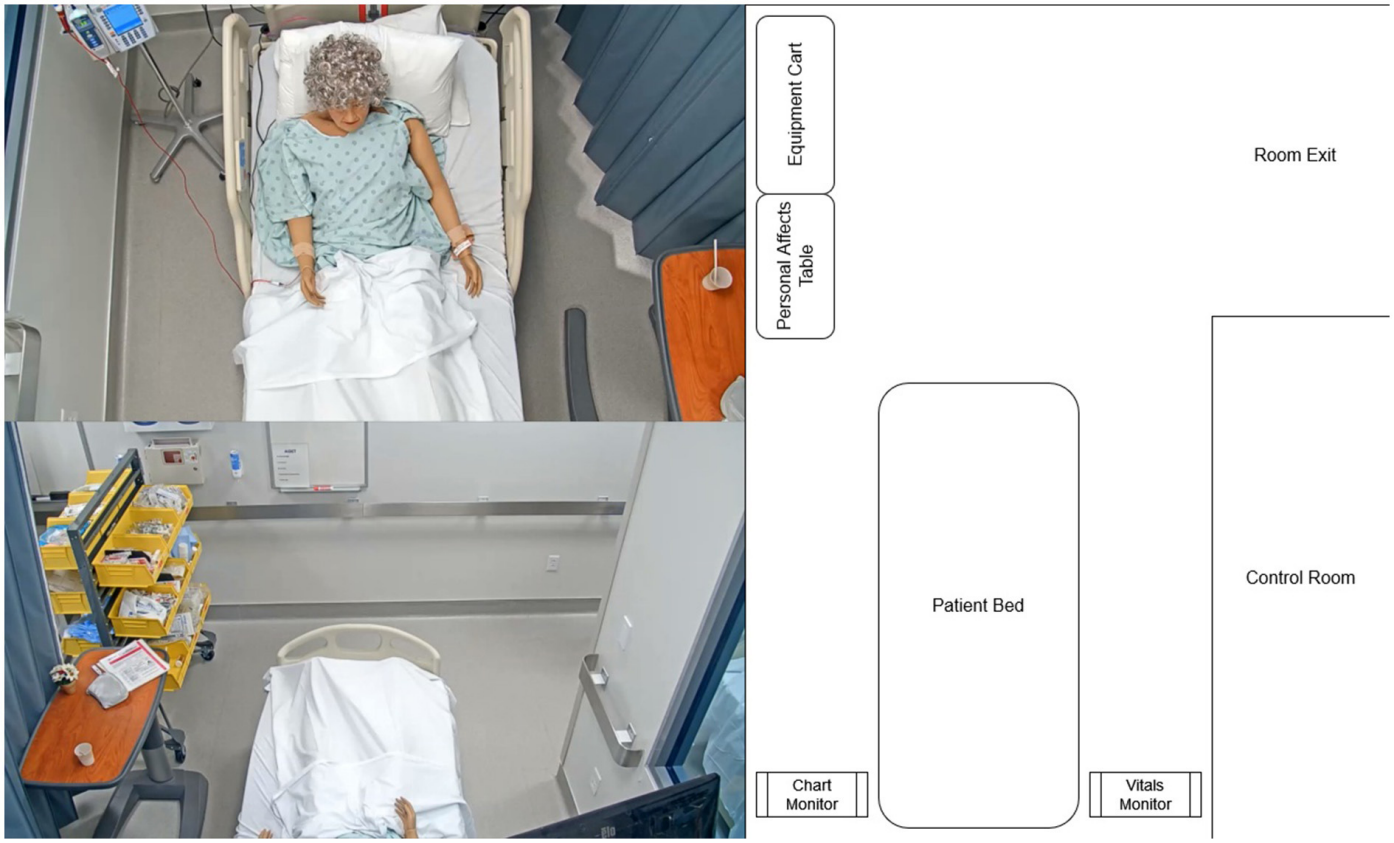
Layout of the simulated hospital room from three viewpoints: the head camera (top-left), foot camera (bottom-left), and an abstract map representation (right).

The approach presented in this paper is supported by a case study that analyzes student nurses training in an MRMB environment. The training took place in a simulated hospital room, which was equipped with standard medical equipment and monitors for information display and communication of the providers orders. The patient was represented by a high-fidelity manikin that was exhibiting distress symptoms and a deteriorating health state. The simulated hospital room is displayed in Figure 3. All of the participating students were undergraduate (BSN) level nursing students in their first year and prior to the study had completed one semester of coursework, which included some simulations similar to those studied in this work. The simulations we study in this paper were part of the students' normal coursework requirements, and no changes to the content of the simulations were made by the researchers.

In more detail, the patient manikin is a SimMan 3G advanced patient simulator from Laerdal Healthcare that supports hands-on deliberate practice, development of decision-making skills, and improved communication and teamwork among learners (Laerdal Medical, 2022b). Prior to beginning the training, the basic scenario and simulation is pre-programmed using the Laerdal Learning Application (LLEAP) (Laerdal Medical, 2022a). This allows the instructors and simulation designers to set the initial state (vital signs, physical presentation, eye and chest movements, etc.) of the manikin, as well as a preset timeline of cue-action associations to change the state of the manikin as time progresses and the scenario evolves. For example, the timeline might be programmed to make the manikin's heart rate rise steadily if a nurse does not begin to administer proper medication within 10 min of the start of the training episode.

In addition to these presets created prior to training, an instructor in a control room can modify the patient state in real-time by interacting through the LLEAP software. The instructors watch the simulation from behind a one-way glass partition, allowing them to observe the nurses' activities, conversations, and interventions. Then, based on the nurses' specific actions (or lack of actions), the instructor makes real-time modifications to the simulation on the LLEAP software. The instructor can also talk as the patient through a microphone in the control room, which can be heard through speakers on the manikin. In the current study, which represented an early training exercise in the nursing curriculum, the instructor was closely involved in the progression of the simulation and manikin.

Three groups of eight nursing students participated in the study over 2 days, taking turns playing their assigned roles in each scenario. The primary participant in each instance of the simulation was a nurse performing a routine assessment on a hospital patient, and discovering a condition that required immediate attention and additional interventions. After diagnosing the patient's condition and performing any relevant immediate stabilization, the nurse was required to call the patient's assigned medical provider to confirm an intervention that would alleviate the patient's newly discovered condition.

Students in the group who were not actively participating in a given run of the scenario watched from a live camera feed in a separate debriefing room. After each scenario was completed, the instructors and the participants joined the full group in the debriefing room, and the instructor guided a discussion-based debriefing of the simulation. Each instance of the simulation took between 5 and 20 min, and parameters of the patient's condition were changed between each run to ensure the next set of students did not come into the scenario with full knowledge of the condition and the required intervention.

All students who participated in the study provided their informed consent. With this consent, we collected data using multiple sensors: (1) video data from two overhead cameras that captured the physical movement and activities of all agents in the room (nurses, providers, and the manikin); (2) audio data also from the camera videos that captured the nurse's dialog with the patient and the provider; (3) the simulation log files that tracked all of the patient's vital signs and data from the sensors on the manikin. In addition, a few students, who provided a second informed consent on collecting eye tracking data, wore eye tracking glasses that allowed us to record their gaze as they worked through the simulated scenario. The study was approved by the Vanderbilt University Institutional Review Board (IRB).

In this paper, we chose two of the recorded scenarios for our case study, in both of which the primary participant wore the eye tracking glasses. In the first scenario (S1), the fictitious patient, Patrice Davis, is receiving an infusion of blood after a bowel resection surgery the night prior. The patient called the nurse stating that she is not feeling well. The goal for this training scenario is for the nurse to assess the patient and diagnose that the wrong blood type is being administered to the patient. The intervention requires the nurse to stop the current infusion and call the provider to discuss further treatment. The primary participant in S1 was a 23 year old female nursing student.

In the second scenario (S2), the same fictitious patient, sometime later in the day, again calls the nurse complaining of pain in the right leg, stating that yesterday “it wasn't bothering me that much but today the pain is worse.” The goal of this training exercise is for the nurse to assess the patient and diagnose a potential deep-vein thrombosis (blood clot) in her right leg. The intervention requires the nurse to call the provider for updated treatment orders and to schedule medical imaging for the patient. The primary participant in S2 was a 24 year old female nursing student.

### 4.2. Cognitive task analysis for learner behaviors

**Figure 4:**
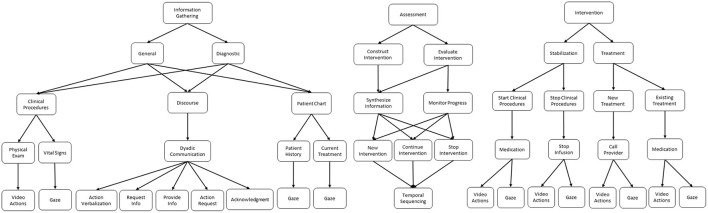
Cognitive task model for the nursing simulation domain.

Using the cognitive task analysis methods previously described, we generated a comprehensive task hierarchy for the nurse training domain. This hierarchy is illustrated in Figure 4. At the highest level of the task model, the overall task breaks down into three primary subtasks: (1) *Information gathering*, (2) *Assessment*, and (3) *Intervention*.

Information gathering represents the processes nurses apply to retrieve new information and monitor ongoing concepts. This process can be further characterized as either *general* or *diagnostic*. In general information gathering, nurses collect non-specific patient and situational information that they use to generate an overall mental model of the patient state. The information collected in this phase is largely standardized for each patient; for example, vital signs are often collected to give a broad overview of patient health. The mental model generated during this phase then leads the nurse to the diagnostic information gathering phase, where the nurse collects more pointed and specific information in service of diagnosing a specific issue with the patient. For example, if dialogue during the general information gathering phase reveals that the patient is experiencing leg pain, then the nurse might follow-up with a physical examination of the leg during the diagnostic phase in order to gather more specific information about the issue.

Assessment represents the processes used to synthesize gathered information in order to construct and evaluate specific solutions and interventions. In addition, we further decompose assessment into intervention *construction* and intervention *evaluation*. During construction, nurses synthesize and combine the information gathered from the environment to generate an intervention that represents a plan of action(s). By drawing on their prior knowledge of patient health and clinical procedures, and their current mental model of this specific patient established from the gathered information, nurses differentially construct a plan for how to help the patient.

During evaluation, similar processes are applied to synthesize information, but this time with a further emphasis placed on monitoring the progress of patient health over time. Temporally, the evaluation phase typically takes place after the nurse has already intervened in some way, and serves as a method to verify that progress toward the intervention goals is being achieved. The evaluation results in one of two possibilities depending on whether progress is made: either continue the intervention further or stop the intervention and re-assess to establish a new plan.

Intervention represents the actions and processes that nurses take in service of a specific goal related to patient health. These interventions are characterized as either *stabilization* or *treatment* procedures. During stabilization, the goal of the nurse is to fix any immediate threats to patient health. For example, in scenario S1 of our case study, the nurse typically turns off the infusion of blood, so that no further harm comes to the patient because of the incorrect blood type infusion. This action does not actually solve the underlying problem, i.e., the patient requires a different blood type infusion, but rather represents mitigation of an immediate threat before treatment of the underlying problem can begin. As a second example, if a patient were to stop breathing, the nurse would typically start resuscitation procedures. Here again, these resuscitation procedures do not fix the underlying cause of the patient's condition, but rather stabilizes the patient back to a point where they are not in immediate danger so that treatment of the underlying condition can begin.

In the treatment phase of intervention, the nurses' actions are in service of fixing underlying health issues that could cause danger to the patient's health in the future. For example, a nurse might start administration of chemotherapy drugs for a cancer patient. In this case, the medication is not designed to help immediate symptoms, but is rather part of a longer term plan to fix the underlying condition and put the cancer into remission. During the treatment phase, nurses will either start/continue an existing treatment order if they are aware of the patient's condition and a provider has prescribed the treatment. If the nurse finds a new condition in the patient, they will contact a provider to follow-up and get a new treatment order.

### 4.3. DiCoT analysis

**Figure 5:**
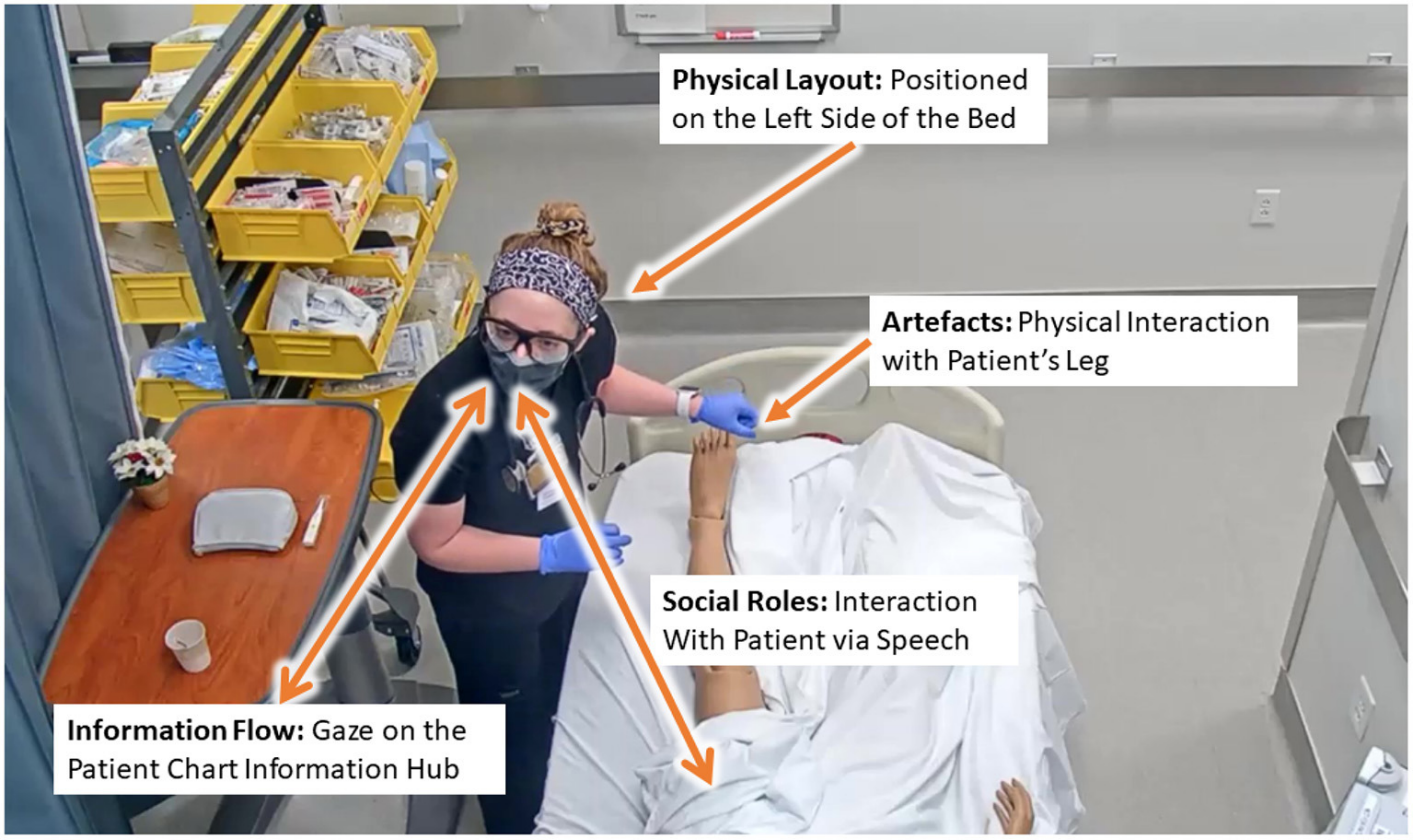
Example of the distributed cognition in the context of nurse training across the physical layout, artifacts, and social themes.

As discussed, the DiCoT framework with its five themes: (1) physical layout, (2) artifacts and environment, (3) social structures, (4) information flow, and (5) temporal evolution; provides a qualitative framework for analyzing learner activities in the training environment. Results from this qualitative analysis then provides the basis for analyzing the multimodal data and inferring nurse activity and behavior information with supporting context. Figure 5 illustrates this idea in context. In this example, the nurse distributes her cognition across all five of the themes:

1. Physical layout by her position on the left and right sides of the bed;
2. artifacts and environment by her physical interactions with the available instrumentation and patient manikin;
3. Social roles by her verbal communication with the patient manikin;
4. Information flow by her referencing of the patient chart monitor; and
5. Temporal evolution by following the sequence of her actions in the environment over time.

Using the five themes and 18 principles (see Table 1), we performed a DiCoT analysis of our nurse training simulation scenarios. We discuss our analysis for each the five themes in greater detail next. Similar to the analysis in Rybing et al. (2016), references to the specific principles are listed parenthetically as they relate to the description of each theme. For example, (P1) refers to Principle 1, i.e., Space and Cognition.

#### 4.3.1. Physical layout theme

The complete layout of the room from three viewpoints can be seen in Figure 3. For the remainder of the paper, when discussing physical positions we will describe the positions in reference to the map view shown on the right-hand side of this figure. For example, *left of the bed* describes the area on the left-hand side of the map containing the patient chart and personal effects tray, while *foot of the bed* describes the area at the top of the map containing the equipment cart and the doorway.

The overall physical layout covered in the simulation environment mimics the layout of a typical hospital room, where the trained nurses apply their learned skills on real patients (P3, P17). In the center of the room along the back wall is the patient bed, where the manikin is placed. To the right of the bed is a computer monitor that displays the vital signs of the patient as graphs (P2, P7). The default graphs and other vital displays are large enough so that the nurse can see them from any position in the room (P5, P6, P7), but the nurse can physically interact with the monitor to test certain vital signs and to get more information when she is on the right side of the bed (P1, P5, P6, P7). To the left of the bed is a second computer monitor that displays the patient's chart. The information on this chart is displayed in smaller text font, so the nurse has to be close to the screen to read information and needs to scroll on the screen to view all of the information. In other words, the nurse must move to the left side of the bed to access this chart (P1, P5, P6, P7). Past the foot of the bed, the room opens into a larger area that contains a cart of medical supplies that may be needed to perform clinical procedures (e.g., gloves, masks, needles, tubing, etc.) (P7). Finally, outside of the room is a medication dispensary; the nurses must leave the room and walk to the dispensary to retrieve needed patient medications (P5, P6, P7).

Given the physical arrangement of the room, we divided the physical space of the simulation into four regions that nurses may move between: (1) left of the bed, (2) right of the bed, (3) foot of the bed, and (4) outside the room. As discussed, each of these regions has available equipment and information that the nurses can use to accomplish their goals. Therefore, they may have to move between the regions to achieve specific goals. At the right side of the bed, nurses can perform clinical procedures, such as taking vital signs or interacting with other stationary equipment (e.g., IV pump, oxygen unit). These clinical procedures are components of the *information gathering* or *intervention* tasks in the cognitive task model.

At the left side of the bed, nurses can primarily perform *information gathering* tasks, such as looking at the patient chart or using the phone in the room to call medical providers. However, when on the left side of the bed, nurses may also cross-reference information from the vitals monitor that is on the right side of the bed (P1). This sort of cross-referencing is often accompanied by subtle body movements, for example, deictic gestures that involve pointing at the screen (P4).

The foot of the bed acts as a transition area for high-level cognitive tasks and lower-level sub-tasks. The training nurses enter the room through this area, establish their current goals, their observation (P6) and their situational awareness (P7) in relation the patient in the room. The nurses pass through this region when moving from the left side of the bed to the right (and vice-versa), while gathering information and making decisions on what clinical procedures to perform (P1). They often pick up equipment from the cart along the way (P7). Nurses also have to pass through the foot of the bed to visit the medication cart, or otherwise exit the room. When doing so, the foot of the bed provides a final moment of situation awareness before their horizon of observation shifts and they are no longer directly viewing the room (P6, P7).

#### 4.3.2. Artifacts and environment theme

Within the simulation environment, the actors, in particular the nurses, utilize a variety of artifacts to support their training activities that are outlined in our task model. The first set of artifacts comes primarily in the form of medical equipment; some of them appear in Figure 3, and several have been discussed in previous sections of this paper. This equipment is designed to mimic the look and feel of a real hospital room, serving the primary goal of the simulation to gain transferable skills (P17), while also providing an interface into the patient data and a means for conducting procedures on the patient. Therefore, the medical equipment serve primarily as mediating artifacts (P15), which transform measurements, such as the vital signs of the patient into textual and graphical information that can be interpreted by the nurses (P9, P15).

Another important artifact in the simulation is the script, which is a set of guidelines set by the instructor about the unfolding of events in the scenario. The script outlines the initial conditions (e.g., the patient's condition, expected vitals at start), as well as a set of behavioral triggers (P14) for how the scenario should evolve given the potential actions (or lack of actions) performed by the nurse. For example, the script might specify that if the nurse does not begin infusing medication within 3 min after the scenario begins, the patient's blood pressure will drop. These scripts' trigger factors mediate the temporal evolution of the simulation (P15, see Section 4.3.5)

The manikin, representing the human patient, is another important artifact for the simulation. It provides an interface for the instructor to construct and guide the evolution of the scenario. The manikin is programmable; therefore, the instructor can digitally set parameters for the patient manikin (e.g., vital signs, movements, and conversations), which are then physically enacted by the manikin system (P13). During dialogue between the nurse and patient, the instructor speaks as the patient through the manikin offering additional information to the nurses (P10), as well as instructional scaffolding (P16), when needed. For example, if the nurse fails to take the patient's temperature, the instructor might scaffold this behavior by making a remark through the patient, such as “I also feel a chill,” which may prompt the nurse to check for a fever by taking the patient's temperature. These dialogue acts can also be used by the instructor to evaluate the nurse's understanding and thought processes. For example, consider the dialogue sequence from S1 shown in Table 2. In this case, the nurse has concluded that the blood transfusion is causing the patient's issues, but in order to verify the nurse's understanding, the instructor asks a clarifying question through the manikin.

**Table 2:** Sample dialogue from S1 demonstrating evaluation of the nurse.

1	Nurse:	I'm going to stop this infusion really quickly.
2	Patient:	Why?
3	Nurse:	Because when we give red blood cells, an indication that you're having a reaction to it is low back pain and feeling itchy. So it sounds like you're having a reaction to the blood transfusion.

#### 4.3.3. Social structures theme

Within SBT, there are three main types of users (Rybing, 2018). First, learners (or participants) represent those who participate in the simulation with the purpose of learning skills or having their performance evaluated (Meakim et al., 2013). Second, instructors (or teachers) are those who participate in the simulation with the purpose of directing the simulation to produce learning outcomes for the learners (Meakim et al., 2013). Finally, confederates (or embedded participants) are those who participate in the simulation with the purpose of enabling or guiding the scenario in some way (Meakim et al., 2013). The social structures of the simulation can be derived from the three basic user types of SBT.

In our nursing case-study, each instance of the simulation has three basic users: two students and the instructor. The students act as learners in the simulation, one taking the role of the primary nurse and one taking the role of the medical provider. The instructor takes a dual role as both the teacher as well as a confederate playing the part of the patient. The patient is enacted through the manikin mediating artifact described in the previous section.

#### 4.3.4. Information flow theme

The primary goal for the nurse training in the MRMB simulation is to collect sufficient information about the patient (i.e., the *information-gathering* task) in order to make a diagnosis of the patient's condition (i.e., the *assessment* task). Then, the nurse has to act to alleviate the patient's discomfort and attempt to improve their health state; this is the *intervention* task. Thus, the movement (P8) and transformation (i.e., interpretation) (P9) of information is critical to making the correct diagnosis and conducting the right intervention. There are four primary sources of information in the simulation that follows the general structure of the *information-gathering* sub-tasks in the task model (Figure 4).

The first information source is the primary nurse, who typically provides information in the form of clinical knowledge that is previously learned during schooling and from prior experiences. This clinical knowledge includes

- Declarative knowledge, e.g., what is the nominal range for blood pressure?
- Procedural knowledge, e.g., how does one measure blood pressure accurately?
- Inferred associations using prior knowledge and observed information, e.g., given that the measured blood pressure is greater than normal, does it explain the conditions that the patient is experiencing?
- Diagnostic inferences, e.g., What may be the cause(s)?

It is important to note that the above is considered to be prior information, and not included as an element of *information-gathering* in the task model. Instead it is looked upon as a fixed input to the simulation system. The nurse may be required to recall this knowledge during the simulation, but this recall may not require any form of enactment and interaction in terms of a specific information gathering task within the training scenario.

Next, the patient's electronic medical record (EMR), also known as the patient's chart, is an information source containing a comprehensive history of the patient's prior symptoms, conditions, and treatments. The chart acts primarily as an information hub (P10), which allows the nurse to quickly reference the patient's history in a comprehensive way. However, it also plays the role of a mediating artifact (P15), since the chart is generally divided into sections allowing the nurse to access the relevant historical information related to the current diagnosis task. Additionally, since the chart contains notes from previous nurse shifts and the treatment being currently administered to the patient, it also helps the nurse trainee to better analyze the patient's trajectory and current condition, and use this to determine their goals and the tasks they need to perform (P17).

Third, the nurse is able to perform clinical procedures on the manikin and gather information about the patient's health conditions. These clinical procedures take a variety of forms, but the most common is collecting and characterizing the patient's vital signs. Nurses make use of the clinical equipment as mediating artifacts (P15) to make measurements on the patient and assess their condition. The mediating artifacts transform measurements into textual and graphical information for easier interpretation by nurses and other providers (P9). The output information is aggregated and displayed on the vitals monitor (see Section 4.3.1), which then acts as an information hub (P10). Other clinical procedures can also be performed by the nurses as needed. For example, if a patient is having pain in one of their legs, as in S2, the nurse might perform a physical examination of the patient's leg to gain more information.

Finally, social interactions between the nurse and the patient provide important information that is not measured directly. The instructor speaks through the patient to provide some of this information to the nurse(s). This information often provides elaborations of the patient's symptoms and additional symptoms that are not directly measured. For example, the patient might describe the location, severity, and history of their pain. These social interaction represent the *discourse* sub-task in the task model.

As the simulated scenario evolves, information primarily flows from the four information sources described above to the nurse (P8), who then process the information (P9, P18) and act on it (P14). When nurses enters the room, they generally begin with a brief interaction with the patient, and this results in information transfer about the patient's general conditions and symptoms from the patient to the nurses. This typically provides an initial baseline for the nurses to check for additional symptoms and start making diagnostic inferences (P13). Thus, it is a component of the *general information gathering* sub-task in the task model.

Next, the nurses typically take some time to reference and review the chart, synthesizing the information that they just heard with the patient history before returning to a more extended dialog with the patient to extract more specific information to support diagnostic inferences. The nurses may ask a series of questions to the patient combining what they saw in the chart with their clinical knowledge (P14). This discussion is typically followed-up by one or more clinical procedures, such as taking vital signs and performing physical examinations. This cycle of discussion with the patient followed by clinical procedures can then be repeated as necessary until the nurse reaches some form of conclusion about the patient's condition. At a higher-level, this can also be thought of as a cycle between the *diagnostic information gathering* and the *synthesize information and construct intervention* sub-tasks in the task model.

Up to this point in the simulation, nearly all of the information has been flowing in from the other information sources in the environment to the nurses (P8). However, once nurses collect sufficient information to reach a conclusion, the process reverses and the synthesized information and resulting conclusions are provided back to the rest of the system through their resulting actions. Common actions at this point include explaining the situation to the patient, starting and stopping certain treatments (e.g., medications), and calling the medical provider to give an update and request updated treatment. These actions and the general flow of information from the nurse to the environment is an enactment of the *intervention* task in the task model.

#### 4.3.5. Temporal evolution theme

The simulation evolves over time in one of two possible ways: through nurse actions or nurse inaction. The instructor has a script artifact which outlines a set of behavioral triggers that detail how the scenario should change (P14). Most of this script deals primarily with triggers due to nurse inaction. For example, the script might dictate that if the nurse does not start medication within 5 min of the scenario starting, then the patient's heart rate begins to climb steadily. On the other hand, scenario changes due to nurse actions are primarily dictated by medical and social responses based on the judgement of the instructor (P3). The nurses gather information to evaluate the situation. Then, based on their evaluations, the nurses intervene to alleviate the patient's conditions. Based on that intervention (or lack thereof), the instructor modifies the scenario. If the intervention was correct, then the patient improves and the simulation ends, but if the intervention was incorrect, then the instructor may further decline the patient's health and the nurse must re-evaluate the presented information and try a new intervention strategy. The temporal evolution of the simulation is built primarily along this cycle of information gathering and intervention.

### 4.4. Computational framework

One of the primary goals of this work is to show how quantitative data can enhance the qualitative DiCoT analysis and integrate this analysis with task modeling framework to better analyze and interpret learner behaviors. To do this, we create a computational framework that takes the raw data collected from the different sensors, maps it onto specific features derived from the DiCoT analysis and then interprets them using the task hierarchy. In our case study, we perform analysis on two raw data sources,

1. Overhead video cameras, and
2. Eye tracking glasses.

**Figure 6:**
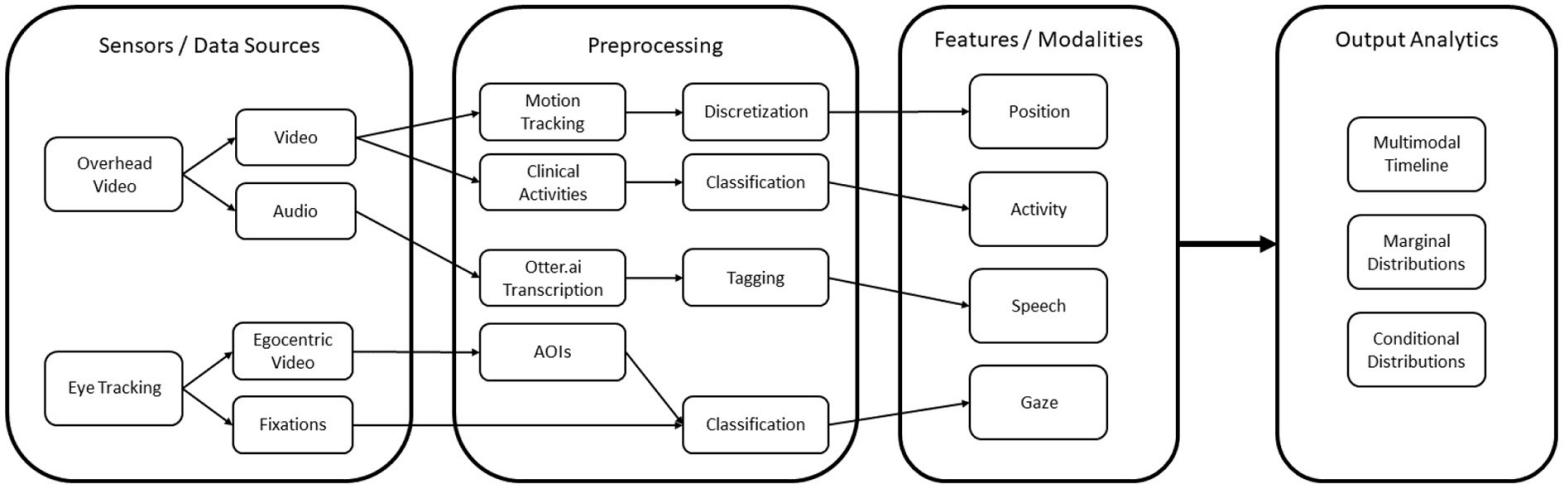
The overall computational architecture used for the quantitative analysis.

These map onto four feature modalities that form the basis of our analyzes: (1) position, (2) action, (3) speech, and (4) gaze. The complete computational framework is illustrated as a block-diagram in Figure 6.

From a combination of the four feature modalities, we construct a complete progression of activities and events on a *timeline*. A complete timeline for the case study analysis of scenario 1 is shown in **Figure 9**, and a similar timeline for scenario 2 is illustrated in **Figure 10**. This timeline structure forms the basis for a second level of analyzes, where information from the extracted features across the different modalities are combined to extract patterns. By combining the aligned features, extracted across the different modalities, we can extract activity information in context, and propagate the low-level actions up the task model to generate inferences about the nurse(s) cognitive processes and their behaviors. We provide a descriptive account for our analyzes of each of the modalities in the subsections that follow.

#### 4.4.1. Position modality

The nurse's positions in the simulated hospital room are derived using visual object motion tracking techniques applied to the video from the two overhead cameras. Our motion tracking techniques are derived from the tracking-by-detection paradigm, which is a two stage approach to tracking Sun et al. (2020). First, in each frame of video, deep learning-based object detection models localize people that appear in the video frame and represent them with bounding boxes. After this detection step, the detections are merged together frame-by-frame into a timeline based on a matching algorithm.

In our case studies, we use the matching cascade algorithm originally developed in Wojke et al. (2017), and later refined for static cameras by Fu et al. (2019). The matching cascade algorithm matches bounding boxes and tracks between subsequent frames based on the distance between the two bounding boxes and approximation of the velocity of the object in the track. In addition, the matching cascade algorithm matches the bounding boxes iteratively based on the age of a detection and track, leading to lower false positive rates.

However, these motion tracking techniques only produce a track of the nurses in reference to the video frame. We need to map these tracks into the nurses' positions in the hospital room as we have described in the physical layout theme of our DiCoT framework. To accomplish this, we extend our traditional motion tracking techniques to project the camera-space motion tracks onto a top-down map representation of the environment (see Figure 3, Right).

Our approach for mapping these camera-space tracks onto this hospital room space computes a planar homography, which associates known points in the camera-space to known points in the map-space using rotation, translation, and scaling operators. Given the computed homography matrix, we can project the camera-space tracks onto the room-space for each frame of video, using the center of the person's bounding box as the projected point. This results in a continuous time-series of nurse positions in the simulation room relative to the top-down map. Further details of this map-projection object tracking can be found in Vatral et al. (2021).

While the continuous time-series of nurse positions in the hospital room is a useful analysis tool, on it own, it lacks the semantic context necessary for meaningful insights. To add this semantic context back to the position data, we discretize the continuous positions into four regions developed using DiCoT analysis (see Section 4.3.1): (1) left of the bed, (2) right of bed, (3) foot of the bed, and (4) outside the room. To perform this discretization, we define a polygonal region on the top-down map of the hospital room for each of the DiCoT semantic regions. Then for each timestamp of the continuous track, we check the polygonal region that contains the nurse's position and assign that label to the given timestamp. This allows us to track in terms of time intervals of nurse positions in the different semantic regions of the room, and when they transition between these regions.

#### 4.4.2. Action modality

In addition to providing position information, analysis of the overhead camera video also provides important information and context for the actions that the nurse performs in the training scenario. Specifically for this case study, we annotate instances in the video where the nurse performs an action by physically interacting with any of the artifacts in the MRMB environment previously identified from the DiCoT analysis.

Additional contextual information can be derived by combining the physical activity that defines an action with other modalities. For example, analyzing speech (see Section 4.4.3) may provide additional information about why a nurse is performing a specific action, or how two nurses are coordinating their actions, for example, when they are jointly performing a procedure. Similarly, a coding of the nurses' gaze (see Section 4.4.4) may provide additional information about how a nurse is performing an action. In some situations, the nurse may look at the same object that they are physically interacting with; in other situations, the nurse may look at a different object than the one they are physically interacting with. As an example, while physically examining a patient, a nurse may turn their gaze to the vitals monitor to see how their current measurement may match with other vital signs (e.g., blood pressure being measured and heart rate of the patient). These examples clearly illustrate the importance of combining information across modalities for action annotation to gain a complete understanding of the nurses' activities in the training environment.

To perform action annotation, we have developed a coding schema based on the artifacts from the DiCoT analysis, which represents all of the high-level objects that nurses physically interact with during the simulation. These objects are primarily medical equipment, e.g., the patient chart, the vitals monitor, and the IV unit. They also include specific parts of the patient that are relevant for physical examination in these scenarios, e.g., the patient's hands, legs, body, and head. In total, we coded nurse actions into 13 categories for the two scenarios in our case studies, which can be seen on the timelines for each scenario (**Figures 9**, **10**). The annotation recorded the action category along with start and end timestamps with a one-half second fidelity. Nurses were considered to be performing a given action category if they were physically interacting with the action object using some part of their body, typically their hands. For example, if the nurse was holding a phone or touching the dial pad, then they were coded as performing the *phone* action. Alternatively, if the nurse's hands were on the mouse and keyboard of the chart computer, then they were coded as performing the *patient-chart* action.

#### 4.4.3. Speech modality

Raw speech is collected from multiple streams that include the audio from the two overhead cameras, and each of the Tobii eye tracking glasses. For this case study, we only analyzed audio from the overhead camera at the head of the bed. In future work, particularly during simulations with a greater focus on teamwork, we intend to analyze audio by creating an egocentric framework for each agent in the training scenario.

While raw recorded speech patterns are useful for some tasks (e.g., emotion detection), most NLP tasks perform analysis directly on a body of text, which requires raw audio to first be transcribed as a preprocessing task. For the current case study, we utilized the Otter.ai speech transcription service (Otter.ai, 2022). After transcription, the speech text is annotated (tagged) with specific events for analysis via the BRAT Rapid Annotation Tool (BRAT) (Stenetorp et al., 2012). Based on the task model (see Section 4.2), we developed a tagging schema for the speech data, which breaks down the dialogue into six speech event tags, which are enumerated below:

1. *Generic, introduction*: Refers to introductory speech such as greetings.
2. *Generic, acknowledgment*: Refers to generic acknowledgments of understanding, typically used as part of closed-loop communication patterns.
3. *Information, request*: Refers to the soliciting of information from another person.
4. *Information, provide*: Refers to the furnishing of information to another person.
5. *Action, verbalization*: Refers to the verbalization and explanation of an action. This verbalization can occur before an action begins, while an action is being performed, or after an action has been completed.
6. *Action, request*: Refers to a request for another person to perform an action, typically taking the form of either a question (e.g., Will you do this?) or a command (e.g., Do this).

**Figure 7:**
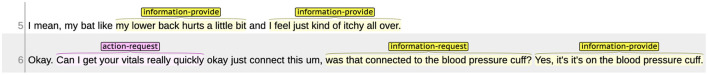
An example of dialogue from scenario 1 which has been annotated using the developed tagging schema.

Figure 7 illustrates a tagged speech snippet from Scenario 1. In this part of Scenario 1, the patient indicates that her “lower back hurts a little bit” and she feels “just kind of itchy all over.” These are examples of the patient providing information to the nurse, so they are tagged as “Information, provide.” The nurse then responds with an “Action, request” by indicating that she (the nurse) would like to check the patient's vitals. The nurse then asks the instructor whether the vital signs device is connected to the blood pressure cuff, which is tagged as another request for information. The instructor then responds to the nurse in the affirmative, which is another instance of “Information, provide.” Notably, the nurse asks “was *that* connected to the blood pressure cuff?” The italicized “that” in this example is ambiguous if speech is the only modality considered for analysis. However, applying the vision and gaze modalities make it clear that the nurse is referring to the device used to actually take the patient's blood pressure. This is an example of how multimodal approaches can augment the information obtained from simulation-based learning environments.

Additionally, it is important to note that there are transcription errors in Figure 7. An important research consideration is whether or not to correct these errors before conducting the analysis. Human-in-the-loop transcription corrections provide the cleanest text to feed into the language model during analysis; however, there is a trade-off. Human-engineered text is expensive to generate time-wise, as manually correcting transcriptions involves reading every piece of a transcribed block of text. With large corpora, this is infeasible. Additionally, human-in-the-loop transcription correction precludes online analysis, as a human would first have to manually edit the transcription before it is used in a downstream task. Lastly, there can be an advantage to having a certain degree of noise in the data, as this can prevent language models from overfitting. Contemporary language models are traditionally trained on large corpora of canonical text. Because speech is rarely canonical, fine-tuning a language model to recognize spoken text is a challenge. However, this can often be mitigated (at least in part) by injecting noise (misspellings, for example) in the data (Cochran et al., 2022). It is for these reasons we decided to annotate the text as-is from Otter.ai, without manually correcting the transcriptions.

#### 4.4.4. Gaze modality

Gaze data is collected using Tobii Glasses 3. The glasses record multiple raw data streams including egocentric-view video, audio, eye gaze (2D and 3D), and inertial movement units (IMU) (Tobii Pro, 2022). The eye gaze data stream is sampled at 50 Hz and contains 2D coordinates corresponding to the egocentric video and 3D coordinates with respect to the camera's coordinate system. The egocentric video is sampled at 25 Hz in 1920x1080 resolution. The IMU sensors onboard the glasses include an accelerometer, gyroscope, and magnetometer, which are sampled at 100 Hz, 100 Hz, and 10 Hz, respectively. Through the combination of all data streams recorded by the Tobii glasses, the nurse's experience in the simulation is logged with high fidelity.

Given the high sampling rate and noise present in eye-tracking data, *fixation classification* is a common practice in the eye-tracking literature to pre-process raw gaze data and prepare it for further analysis (Bylinskii et al., 2015; Liu et al., 2018). Our initial pre-processing step applies Tobii's Velocity-Threshold Identification (I-VT) fixation filter to extract reliable fixation and saccade data. The classification algorithm identifies fixations and saccades based on the velocity of the eye's directional shift and a set of hyperparameters (Olsen, 2012). The default values provided by Tobii for the I-VT fixation filter were used during our analysis (Tobii Pro, 2012).

**Figure 8:**
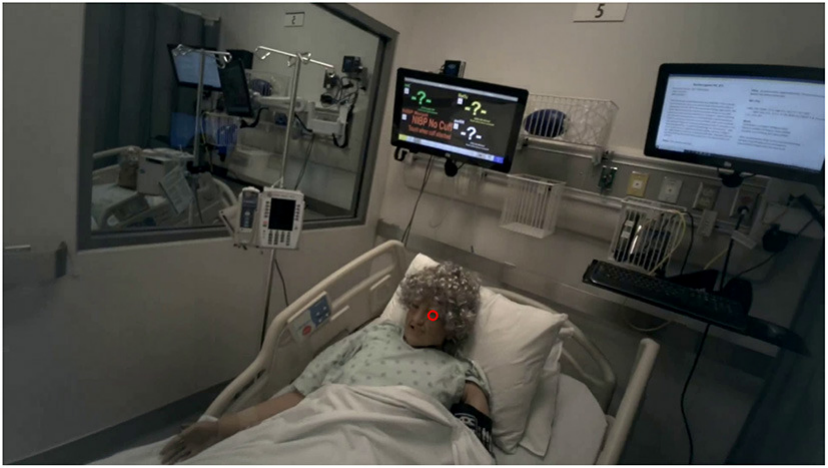
An example of fixation overlay from Scenario 2 used for manual annotation. In this frame, the resulting AOI is “patient”.

The final preprocessing step is to encode the fixation data into areas of interest (AOI) sequences. Linking fixations to AOIs bridges the gap between direct sensory output to domain-specific content, thus providing further insight into the nurses' attention and engagement. The temporal evolution of nurses' visual attention is represented by AOI sequences. In this study, AOI encoding from the fixation data is manually annotated to 11 objects of interest (OOI) that were selected based on the DiCoT analysis (patient, provider, screen chart, paper chart, vitals, medical tray, equipment, keyboard, instructor, one-way mirror, ground). Each of these physical objects are treated as an AOI and are annotated using the egocentric video. The manual tagging is performed through visual inspection of the egocentric video with fixation data overlaid. In each case where the nurse fixates on one of the AOIs, the start and end time of the fixations are recorded. An example of the gaze overlaid on the video is shown in Figure 8, where the red circle marks the fixation.

## 5. Case-study analysis

**Figure 9:**
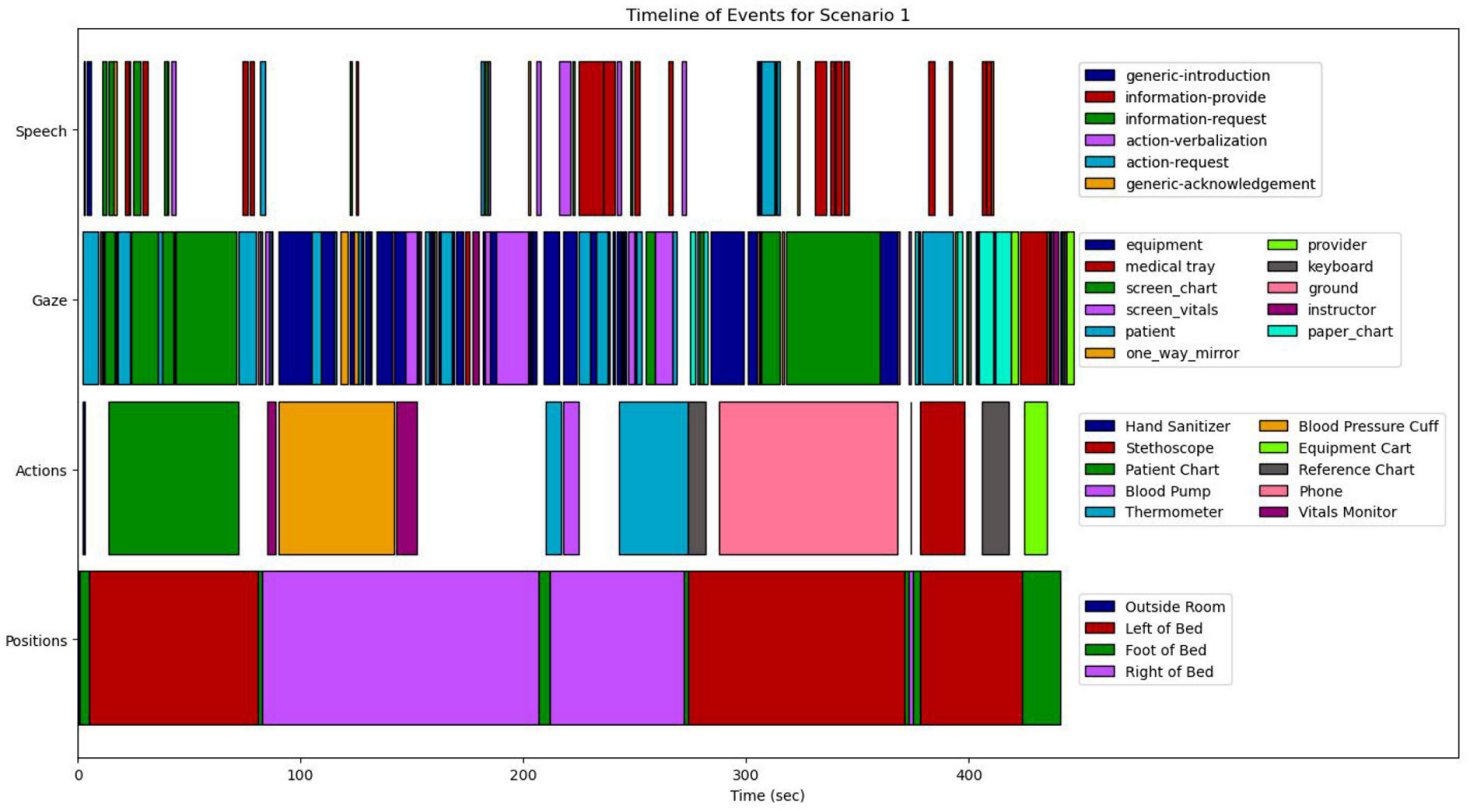
The complete timeline of events for scenario S1 containing annotated data from participant position, action, gaze, and speech.

**Figure 10:**
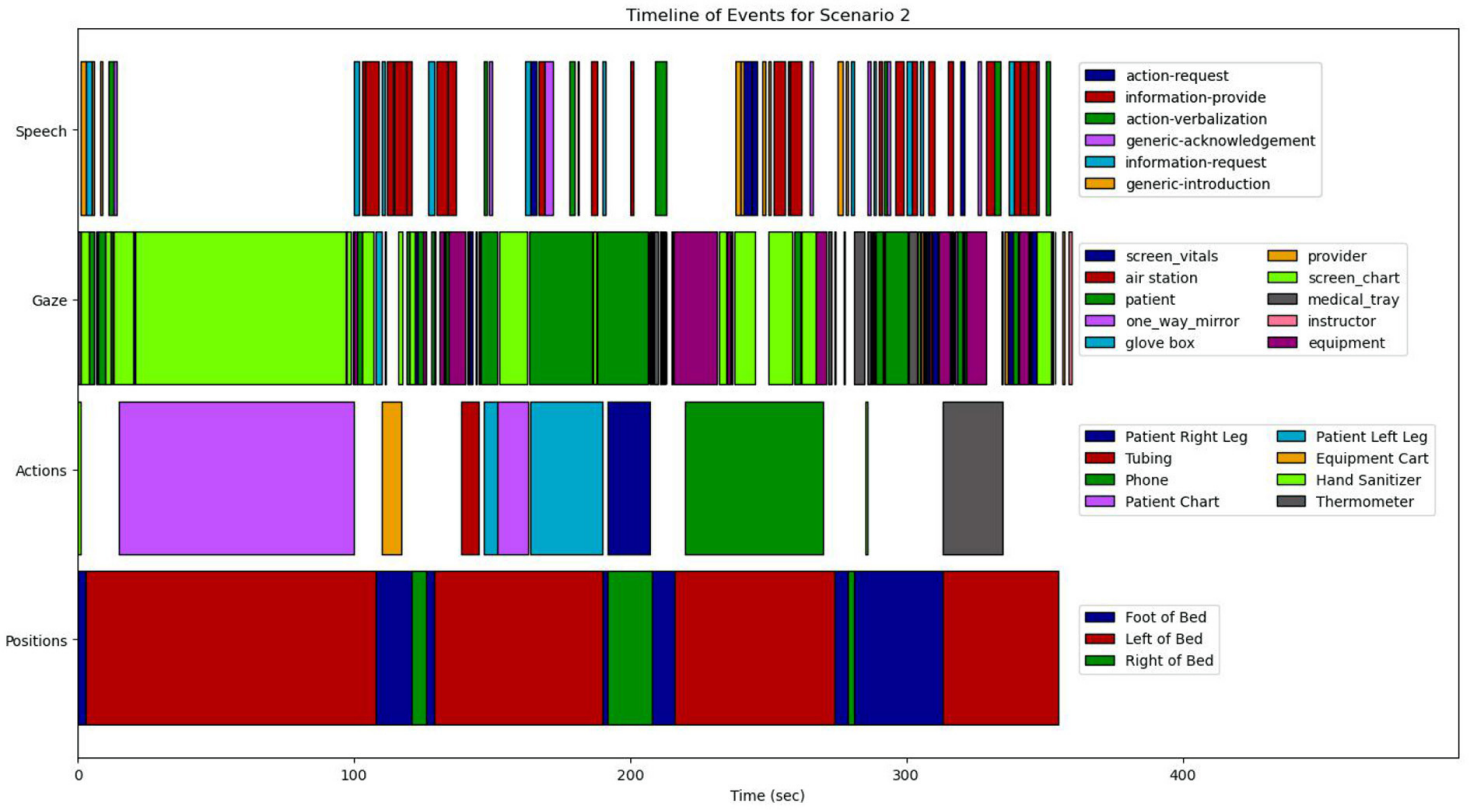
The complete timeline of events for scenario S2 containing annotated data from participant position, action, gaze, and speech.

The alignment and processing of multiple data modalities reveals new inferences about the simulation and the nurse's behaviors. In this section, we analyze and interpret these integrated multimodal timelines (Figures 9, 10) in depth for each scenario. We provide details of the basic breakdown of nurse actions and use the DiCoT framework to interpret these actions in context and map them onto the task analysis hierarchy. In addition, we compare across the two scenarios to see how the nurses differed in their cognition and use of environmental affordances in the MRMB environment.

### 5.1. Scenario 1

For scenario S1, the timeline breaks down into approximately five high-level segments. The first segment follows the general *information gathering* task established by the task analysis model in Section 4.2. During this segment, the nurse first enters the room and greets the patient, and then moves off to the left side of the bed. In this position she begins a period of alternating between reading the patient chart and conversing with the patient, as indicated by her eye gaze moving between the patient and chart monitor. The conversation here is primarily pairs of *information-request* and *information-provide*, indicating that the nurse is asking the patient questions to clarify and expand on the information the nurse is reading from the chart.

Once the nurse decides she has enough information to build her initial mental model of this patient's situation, the simulation enters the second phase. This transition is marked by the nurse moving from the left side to the right side of the bed, as seen in the position modality around 80 s into the scenario. As previously shown from the DiCoT analysis, this movement between regions in the room is an important indicator of task transitions. During this new segment, the nurse moves to the *diagnostic information gathering* phase described in the task model. In this phase, the nurse increases her interactions with the equipment and the vitals monitor. We derive this information from the gaze, which shows movements between equipment, the vitals monitor, and the patient. In addition, her physical actions show interaction with the vitals monitor and the blood pressure cuff. In this segment, we can apply information from the DiCoT framework to provide additional context for establishing these action as diagnostic information gathering. Because of this movement from the left to the right side of the bed (physical layout) and the increased interaction with clinical equipment (artifacts and environment), we infer that the nurse is attempting to establish and refine her diagnostic inferences from the initial information gathering phase. She performs clinical procedures, such as taking additional vital sign measurements to aid her diagnostic hypothesis formation.

In this segment, we also see a reduction in dialogue, which likely has two causes. First, specific to this scenario, much of the information that can be obtained from the patient has already been gathered in the previous segment. Second, the cognitive load associated with performing clinical procedures (e.g., when taking a blood pressure reading) is likely higher than simply reading the patient chart. Because of this, the nurse may focus more on the clinical task at the expense of continuing conversations with the patient. This is especially true for novice trainee nurses who are still learning how to perform clinical procedures in correct and effective ways. Knowing that these clinical tasks require higher cognitive loads and having observed from the control room that the nurse reduced her dialogue, the instructor likely also intentionally reduced their conversations with the nurse during this period. The instructor may have spoken less through the patient while these tasks were being performed to avoid splitting the nurse's attention, conforming to the best practices during SBT (Fraser et al., 2015).

Around 220 s into the scenario, our video analysis shows that the nurse begins to interact with the blood pump, which implies a transition to the third segment of her overall task. According to the DiCoT analysis, the blood pump is a mediating artifact, not an information source. Given this additional context, we can conclude that the nurse has reached the end of her diagnostic information gathering phase and has begun the *intervention* process in this new segment. Since the nurse interacts with the blood pump at the start of the intervention process, we can hypothesize that the nurse has reached a diagnostic conclusion, and suspects the blood infusion process. In other words, the nurse suspects that the patient is being administered the wrong blood type during infusion.

Specifically, in this segment the intervention represents the stabilization process, which requires the nurse to stop the blood infusion and prevent any further damage to the patient's health because of the infusion of the incorrect blood type. At the start of this segment, as our video analysis shows, the nurse stops the infusion, but the speech modality also records an *action-verbalization* event. The speech analysis module interprets the nurse telling the patient that she is turning off the blood infusion. This is immediately followed by the patient asking “Why?,” and the nurse follows up with a proper diagnostic explanation, i.e., “an incorrect blood type is being infused.” This discourse interaction, transcribed in Table 2, is an example of the dual social role of the instructor as both the *teacher* evaluating the nurse, and a *confederate* playing the part of the patient (see Section 4.3.3).

It is quite reasonable for a patient to ask questions about their condition and the treatments being administered in a real hospital setting. The instructor plays this role as the confederate. Indirectly, some of the questioning by the patient (i.e., the instructor as the confederate) also serves as an evaluation of the nurse who must explain her reasoning. This sort of evaluation questions arise from the instructor's role as the teacher, rather than the confederate. Since the instructor is playing both social roles, this discourse interaction may fulfill multiple pedagogical roles in the simulation scenario, i.e., how the nurse conveys diagnostic information to the patient to reassure them, and how the nurse has combined all of her observations to make diagnostic inferences. In this same time interval where the nurse interacts with the blood pump and verbally explains what she is doing, we also see her gaze moves between the equipment (the blood pump) and the patient, which is likely part of the social dynamics when interacting with a patient. The nurse should not ignore the patient while performing clinical procedures, which is exemplified here as the nurse shifting her gaze between the patient and the blood pump.

Once the stopping of the infusion is observed in our video analysis, the fourth segment of the simulation begins, with the transition marked again by the nurse's movement; this time the movement is from the right side of the bed back to the left side. This segment maps on to the *treatment intervention* phase of the task model. Since the diagnosed issue is not one that already has a physician prescribed treatment, the nurse calls the provider to make them aware of the new situation, as indicated by the *phone* action around 290 s into the scenario. We can see that during the period where the nurse is using the phone, her gaze is primarily on the chart monitor, likely because she is reading off the patient's information to the provider over the phone. This is also consistent with the speech acts, where we see several sequential *information-provide* acts, again likely because she is giving the patient's information to the provider over the phone.

During this same period, the speech also shows a few *action-request* events, which correspond with the nurse requesting that the provider come to the room to confirm the diagnosis. Shortly after the phone call, the final segment of the simulation begins, marked by the arrival of the provider around 390 s into the scenario. In this segment, we again see several sequential *information-provide* acts in the speech corresponding to the nurse explaining the patient's situation to the provider. The nurse and the provider then look at a reference chart, which contains information about the protocol to re-test blood type. Finally, the two move to the foot of the bed and begin examining the equipment cart, likely to collect the necessary equipment to draw the patient's blood. This marks the end of the training scenario, and the nurse moves on to a debrief session outside of the simulation hospital room.

### 5.2. Scenario 2

In scenario 2, the timeline breaks down into four high-level segments. Once again, the first segment represents the *general information gathering* task. The nurse enters the room and moves to the left of the bed by the patient chart monitor. During this initial movement period, there is a short sequence of alternating *information-request* and *information-provide* speech acts, indicating the nurse asking the patient initial questions to learn about their general background and current condition. Just as in S1, the initial movement to the left side of the bed is a significant indicator of entering an information gathering phase, as indicated by the physical layout DiCoT analysis.

This initial movement and speech is then followed by a long period of attention strictly on the chart monitor, as seen in both the actions and gaze, as well as the absence of any dialogue. As shown in the information flow DiCoT analysis, this chart monitor is a significant information hub in the room and further supports this segment as information gathering. The absence of dialogue here is also particularly interesting when compared to the nurse in scenario 1, who tended to multi-task dialogue with the patient while reading the chart. However, here we see a different information gathering strategy of first spending devoted time to the chart, followed by a shorter period of *information-request* and *information-provide* acts (e.g., question and answer) around 100 s into the scenario.

During this question and answer period, the nurse's position moves quickly between the foot of the bed, the right of the bed, and back to the left of the bed, with her gaze also moving rapidly between pieces of equipment and other artifacts in the room. On its own, it is unclear what exactly the purpose of these rapid movement and gaze changes are; however, given that this occurs while the dialogue is primarily question and answer, which is an information gathering task, it is likely that the movement and gaze are also related to the information gathering. While the nurse is using dialogue to gather information about the patient during this period, she is simultaneously also gathering information about the available equipment and physical layout of the room using her movement and gaze.

At this point, the second segment of the simulation begins, marked by the nurse moving back to the left side of the bed and her gaze now stabilizing back on the patient and chart, around 140 s into the scenario. Like scenario 1, the second segment represents *diagnostic information gathering*. Having determined patient history and the current issue with the patient, i.e., severe right leg pain, the nurse begins a *physical examination* of the patient in order to further refine her diagnosis of the problem.

The nurse begins examining the patient's left leg for a short period of time, while asking the patient whether certain areas that the nurse touches are tender. This is derived from our analysis of nurse's actions, which show physical interaction with the patient's leg, along with speech analysis which shows sequential *information-request* and *information-provide* acts. After this exchange, the nurse turns her gaze from the patient back to the chart, likely because she is surprised when the leg does not hurt to the touch. At this point, the information she obtained from dialogue with the patient and the patient chart does not match with the physical exam of the leg. Because of the conflicting information, the nurse looks back on the chart to recheck the information she previously gathered and her diagnostic hypothesis.

After a few more moments of examination and dialogue with the patient, the patient finally speaks up and says, “It's my other leg that hurts.” At this point, the nurse quickly moves over to examine the right leg, as shown in the action data. There are several interesting points about this interaction. First, dialogue of the patient is another manifestation of the dual social role of the instructor. The instructor is acting as the patient in this moment, but also providing some instructional scaffolding, e.g., that the nurse needs to examine the other leg. By inhabiting this dual social role, the instructor can seamlessly introduce the instructional scaffolding into the simulation scenario by speaking through the patient.

Second, by combining data modalities, we gain a much deeper understanding of the nurse's activities in the training scenario. Because we have the eye gaze information and see that the nurse looks back at the chart, we interpret that the nurse realizes that there is an issue before being corrected by the patient. Pedagogically, this is important because it shows a level of metacognitive awareness in the nurse which we may not have realized otherwise. The nurse looks back on the chart to recheck her diagnostic hypothesis because of the conflicting information she has received that the patient's leg does not hurt to the touch. Without this gaze information, we may have surmised that the nurse had gone down a wrong path, and would need to be corrected on her diagnostic hypothesis. However, her looking back to study the chart and asking questions to the patient made us realize through the analyzes that she was reconsidering her current diagnostic hypothesis.

After examining the right leg, the training scenario transitioned into the third segment, marked by the movement of the nurse from the right side of the bed where she was examining the leg back to the left side of the bed. This movement, around 220 s into the scenario, again highlights the physical layout theme of the DiCoT analysis. In this segment, the nurse began the *intervention process*. No stabilization processes are clinically necessary in this scenario, so the nurse immediately proceeded to *treatment*. Just as in S1, there was no physician prescribed treatment, so the nurse called the provider to update them and get a new treatment order, as indicated by the *phone* action. While on the phone, the nurse's gaze was primarily on the patient chart, with a few instances of looking back at the patient, and simultaneously her dialogue was a series of *information-provide* acts. This gaze and speech in combination indicate that she was reading patient information off the chart to the provider, and filling in additional details based on her observations and gathered information of the patient condition.

Shortly after the phone call, the scenario transitioned into the fourth segment, marked by the entry of the provider into the room and the nurse moving to the foot of the bed, around 290 s into the scenario. In this segment, the dialogue shows a series of sequential *information-request* and *information-provide* pairs, indicating that the provider was asking questions to the nurse and the nurse was answering based on her gathered information and assessment of the patient. During this sequence, the provider asked whether the nurse has gathered patient vitals. After realizing that she did not finish this task earlier, the nurse began to interact with the equipment to complete collecting the vital signs, as shown by the *thermometer* action and the nurse's gaze moving between equipment and the vitals screen. The scenario finished with a short discussion about the next steps for treatment, specifically the scheduling of a scan of the patient's leg, shown by the series of *information-provide* acts in the speech at the end of the timeline.

### 5.3. Cross-scenario discussion

In this section, we combine the analysis across both scenarios to demonstrate how the collected data supports the DiCoT analysis presented previously. For this analysis, we will focus on the three primary DiCoT themes which are typically analyzed: physical layout, information flow, and artifacts and environment. We will examine each of the three DiCoT themes individually and how the data-driven evidence supports the major conclusions from that theme.

**Figure 11:**
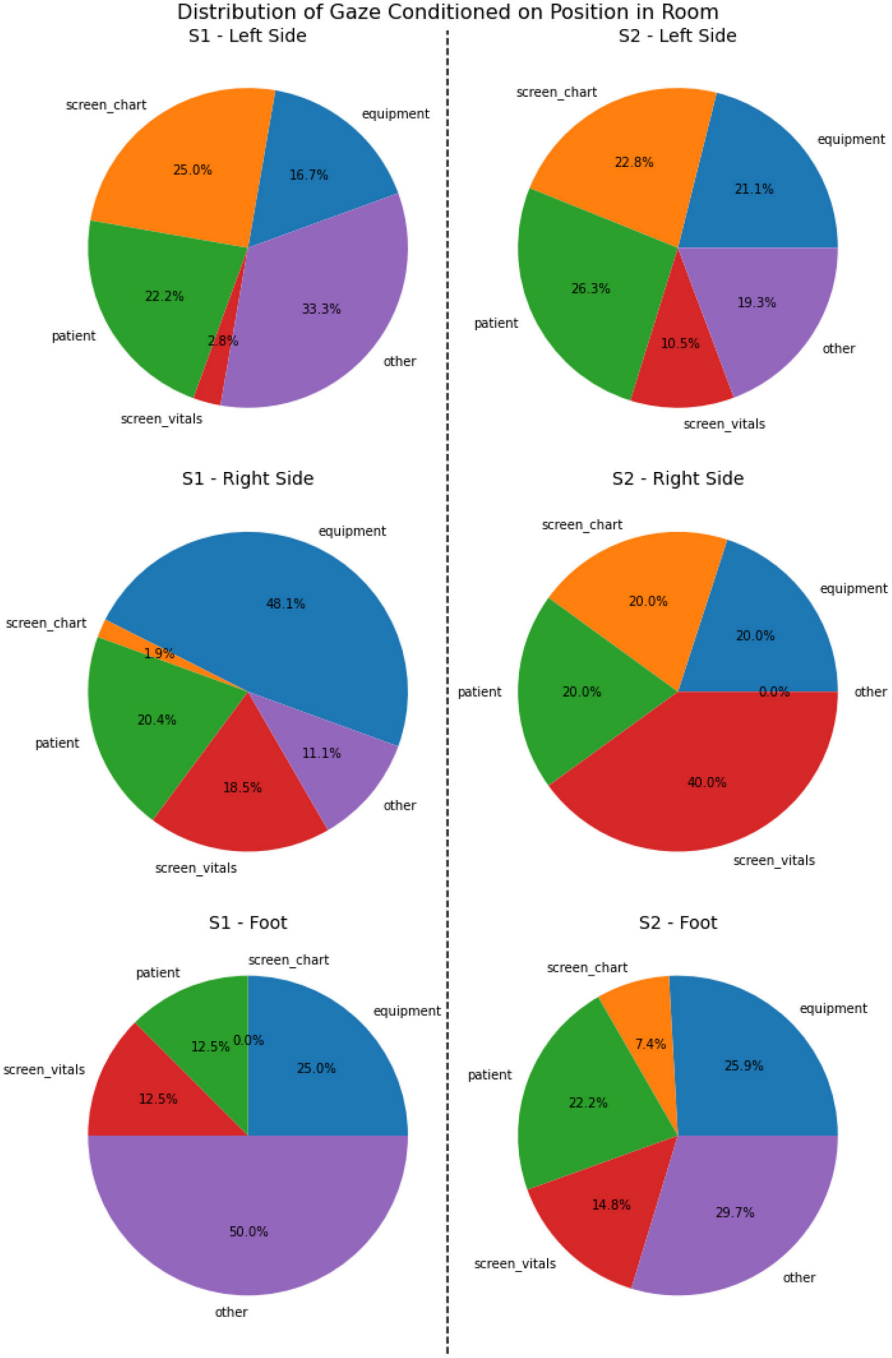
Distribution of gaze across five major object categories conditioned on the nurse's position in the room for each scenario.

**Figure 12:**
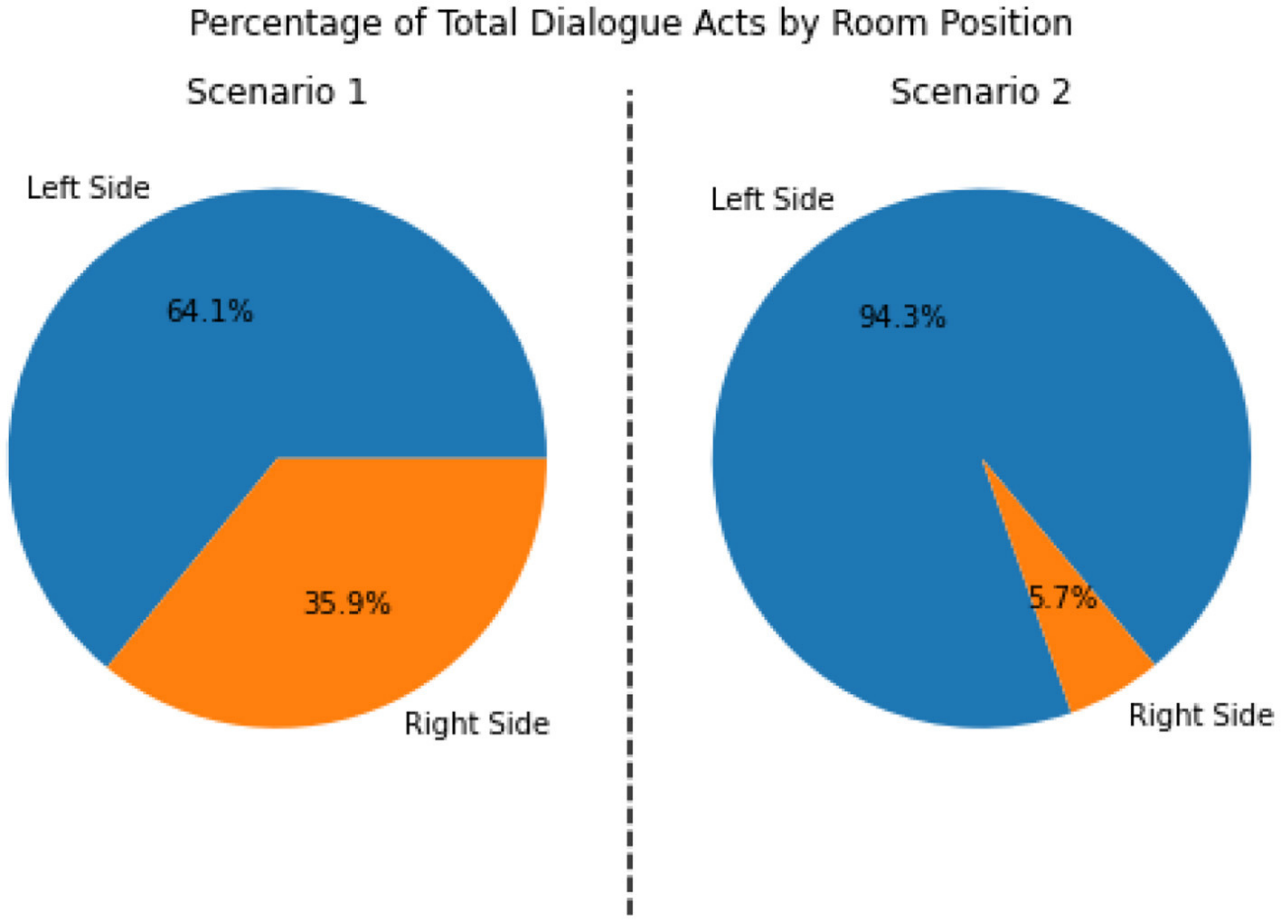
Distribution of total speech acts conditioned on the nurse's position in the room for each scenario.

**Figure 13:**
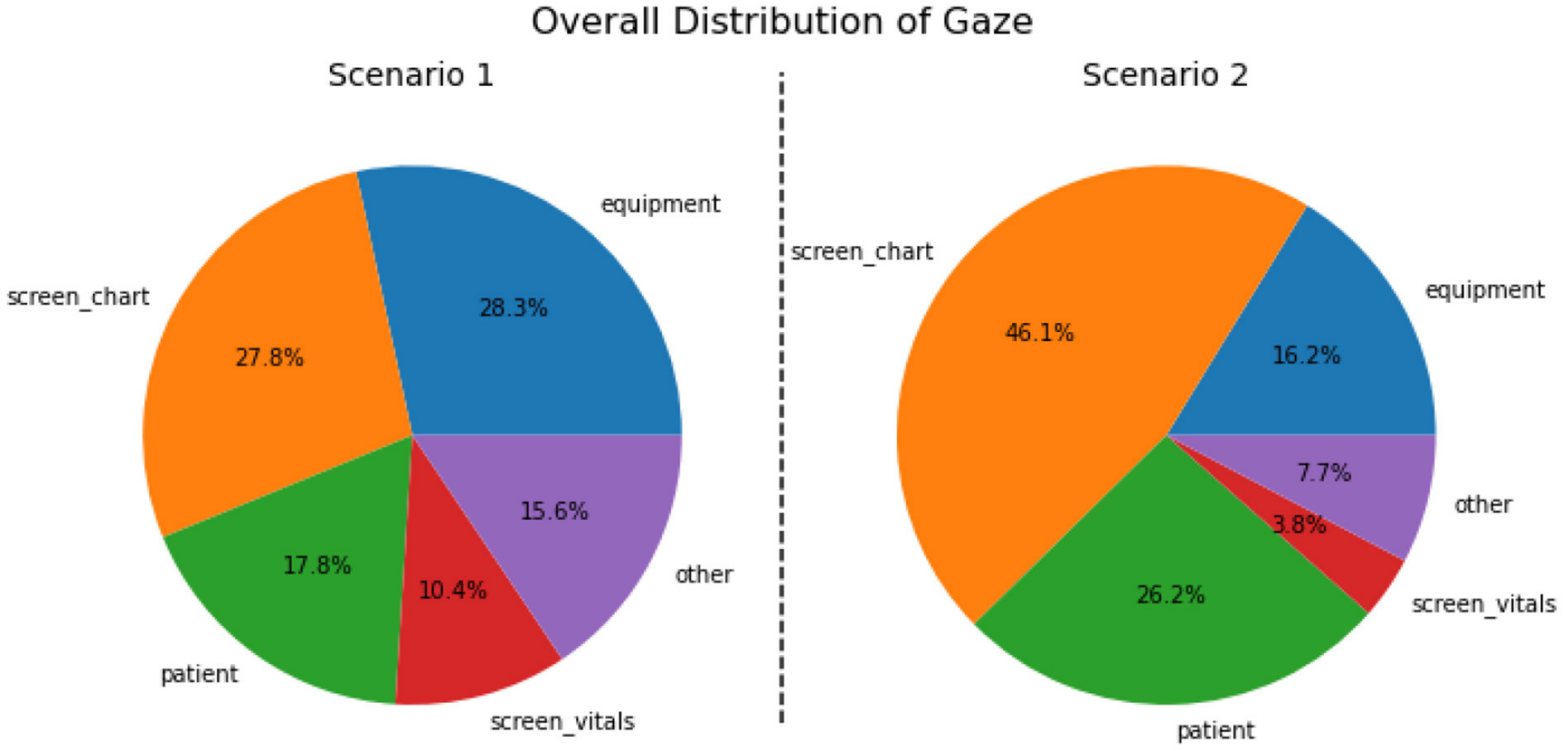
Marginal distribution of nurse gaze across five major object categories for each scenario.

To support the comparison between the contextually different scenarios, we computed a series of marginal and conditional distributions of the four data modalities. **Figure 13** shows the marginal distribution of gaze across the entire scenario; Figure 11 shows the distribution of gaze conditioned on position in the room; and Figure 12 shows the distribution of speech conditioned on position in the room. These distributions were computed based on the modality-aligned timelines (Figures 9, 10) by dividing the sum of the time spent on a given modality class by the total scenario time. For example, to compute the percentage of equipment gaze events conditioned on being positioned on the left side of the bed, we divided the sum of the times spent looking at equipment while on the left side of the bed by the total time spent on the left side of the bed. By comparing the marginal and conditional distributions of the scenarios instead of the scenario timelines directly, we can help reduce the temporal autocorrelation caused by the differences between the scenario contexts. In other words, the distributions provide a more direct comparison between the two scenarios that does not care about the order in which nurses completed actions, since the order is highly dependent on the specific scenario and patient condition.

Beginning with the physical layout theme, a wealth of data supports the roles that space and physical layout play in the nurses' cognition. The timeline analysis shows that both nurses exhibit similar patterns in their movement through the physical space. Each nurse begins by entering the room through the door at the foot of the bed and immediately moving to the left side. The nurses stay on the left side to gather initial information from the chart and conversation with the patient before moving to the right side of the bed to begin their diagnostic clinical procedures. While the specifics of information gathering and clinical procedures differ between the two scenarios, the general movement patterns and associated tasks in these areas of the room remain very similar.

Support for the roles of these spaces can also be seen through the conditional distributions of gaze in Figure 11. For both nurses, the percentage of gaze events focused on the chart and the patient was higher when they were on the left side of the bed, while the percentage focused on the vitals screen was higher when they were on the right side of the bed. This was particularly evident for scenario 1, where focus on the chart and vitals when on the left side of the bed were 25 and 2.8%, respectively. It changed to 1.9 and 18.5%, respectively when they were on the right side of the bed.

For scenario 2, while the difference in gaze for the chart monitor was fairly small, changing from 22.8% on the left down to 20.0% on the right, the difference in gaze for the vitals monitor was still quite large, with 10.5% when on the left and jumping to 40.0% when on the right. These differences between the left and right sides of the bed was also supported by the speech analysis. As shown in Figure 12, the nurses in both scenarios performed most of their dialogue when positioned on the left side of the bed. This suggests that the nurses' information gathering done through dialogue with the patient happened primarily when they were on the left side of the bed. Together, this gaze and dialogue data further confirmed the role of each of these spaces in the room; the left side of the bed was primarily used for information gathering and the right side was primarily used for providing clinical procedures.

For the information flow theme, data from the nurse gaze provided significant support for three primary information sources described in the DiCoT analysis: the chart, the patient, and the vitals monitor. Examining Figure 13, which shows the marginal distribution of nurse gaze over the course of the entire scenario. It is clear that the nurses spent most of their time looking at three primary information sources. Over 50% of the total gaze time in both scenarios was spent looking at these three information sources, with 56% for scenario 1 and 76.1% for scenario 2.

The nurses used these three sources to gather, aggregate, and synthesize information which may have been relevant to the patient's diagnosis and treatment. The timeline analysis also supports the information flow theme, demonstrating the transition from information flowing to the nurse to information flowing from the nurse. In both scenarios, the first two timeline segments involve the nurse gathering information. In the first segment, this information came primarily from reading the patient chart and conversation with the patient. In the second segment, the information came primarily from the nurse performing clinical activities.

At this point, the information flow in both scenarios reversed, with the nurses now becoming the information source and the patients and provider becoming the information recipients. Once the nurses had transformed and synthesized the gathered information, they reported their diagnostic inferences, thereby becoming an information source. In both scenarios, the nurse first provided information on her conclusions to the patient, explaining the diagnosis and how they arrived at that conclusion. Then the nurse provided information to the medical provider, first in the form of general patient information over the phone, and then in the form of explaining the diagnosis once the provider arrived in the room.

Moving on to the artifacts and environment theme, the gaze data again clearly supported the use of medical equipment as the primary mediating artifact. As seen in Figure 13, the nurses in both scenarios spent a significant portion of their time with their gaze fixated on the equipment. In scenario 1, the equipment represented the single highest portion of gaze activity at 28.3%. In scenario 2, the nurse looked at the equipment for less time than in scenario 1, but still for a large portion of the total time: 16.2%, which was third overall in terms of the activities conducted. The difference in time here between the two scenarios can be explained by the context of the patient's presenting issue; in scenario 1, the primary cause was primarily linked to the equipment, i.e., the blood pump infusing the wrong blood type. In scenario 2, the primary cause was internal to the patient. The significant portion of time in both scenarios dedicated to medical equipment is evidence of its fundamental role in the distributed cognition analysis of the training scenario.

Beyond these mediating artifacts, the data also supports the use of several artifacts as information hubs, specifically the chart and vitals monitors. As seen in Figure 13, the nurses spend 38.2% and 49.9% of their time, respectively, looking at these two monitors in scenarios 1 and 2. In addition, the timeline analysis suggests that the nurses frequently returned to these information hubs for confirmation and further checking when they were in doubt about their conclusions. For example, we see this behavior in scenario 2 when the nurse looked back at the chart after her physical examination of the patient's left leg did not support her internal diagnostic hypothesis. This shows that the nurses trust the information provided by these artifacts, which support their cognitive reasoning processes in aid of information gathering and diagnostic reasoning.

## 6. Discussion

Overall, the patterns and distributions derived from our analysis framework clearly demonstrate the effectiveness of our approach in combining qualitative DiCoT analysis with multimodal analytics and the task model to analyze and interpret learner activities and behaviors in the MRMB training simulation. Specifically, this study shows the benefits of our cyclic analysis, with insights generated from both a forward pass of the framework, i.e., using the qualitative analysis to define and structure the quantitative analysis, as well as a backward pass of the framework, i.e., using results of the quantitative analysis to provide more detailed analysis of the learners activities and behaviors than we could generate by pure qualitative analysis, as proposed by the DiCoT framework. The more in-depth information generated by multimodal analysis benefits the two primary stakeholders: (1) learners and instructors through debriefing and after-action reviews, and (2) simulation designers and researchers, who can study the effectiveness of the simulation scripts in promoting effective learning activities. In this section, we discuss the implications of the framework and its resulting insights for both of these groups.

### 6.1. Implications for learners and instructors

The primary goal of any simulation-based training environment is for the trainees to learn, practice, and develop expertise in skills that transfer to the real task environment. In our nurse case-study, this means that the nurses develop new knowledge and experience that supports both the psychomotor skills and cognitive and metacognitive processes. One of the critical components that mediates this knowledge gain, especially for novice learners, is effective feedback mechanisms during simulation debrief (see Section 2.1). It is the analysis of nurse performance and the generation of relevant feedback linked to the performance, where our current work is most likely to impact learners and their instructors in constructive ways. By using our analysis framework to generate evaluations of learner behavior, we can present these insights back to learners and instructors during debriefing (also known as after-action reviews) to help promote constructive discussion among the trainees and instructor as part of a larger formative feedback system.

This paper represents an initial step toward analyzing learner performance and behaviors, and then generating formative feedback, and as a result, this case-study analysis was performed *post-hoc*. Therefore, no feedback was generated for learners. However, with continued research, we hope to develop a formative feedback framework with input and support from the instructors to support effective learning of skills and decision making processes. For example, at the beginning of each scenario in our case study, the nurses both start with talking to the patient and reading the patient chart. However, the ways in which these two actions are sequenced differ greatly between the two nurses. In S1, the nurse tended to multi-task combining dialogue with the patient and reading the chart. On the other hand, in S2, the nurse spent a long period of time solely focused on reading the chart without any interaction with the patient, Only after she had reviewed the chart in some detail, did she start talking to the patient in depth. By generating analytics about the nurses' gaze and speech patterns, we can highlight this difference between the nurses and present this feedback as a discussion point during debriefing: Was there a good reason for the difference in approach between the two nurses? Is it not important that the nurse to communicate with the patient sufficiently often so patients do not feel that they are being ignored? Therefore, some level of multi-tasking may be a useful protocol to adopt at this stage of examining the patient and collecting information about their situation. As a next step, we hope to get nursing instructors and experts in as part this discussion. This will help us generate appropriate feedback that will help learners, and also help instructors in setting up constructive discussion among the learners by presenting contrasting cases (Bransford and Schwartz, 1999).

While this is only one simple example, it demonstrates the underlying concept: analytics generated using our activity analysis framework can be presented back to learners and instructors to help promote meaningful discussion, especially around topics that may be otherwise difficult to identify in a single viewing of the scenario. The design of formative feedback that is actionable and important for discussion is a large research questions in itself (Jørnø and Gynther, 2018; Pardo, 2018) and is beyond the scope of this paper; however, analysis framework we present here represents an important first step in this direction for SBT and MRMB training environments.

### 6.2. Implications for simulation designers and researchers

Because of the cyclic nature of our analysis framework, the insights generated from our analysis and future analytics methods can be used to help refine the qualitative models of the simulation system. This is of particular importance and interest to simulation designers and researchers, as it uncovers new insights to improve our understanding of both the given simulation system and the science of simulation-based training as a whole.

For example, the multimodal data analysis permits the discovery of latent relations between different aspects of the distributed cognition system. In our nursing case-study, this is exemplified through the use of information hubs. The distribution of gaze conditioned on position reveals new insights about the use of information hubs. Initially, the DiCoT analysis revealed the dependency of physical space as a mediator in collection and analysis of the information provided on the two screens as information hubs (i.e., the patient chart and vitals). By combining the physical, artifacts, and information flow segments of DiCoT analysis, we derived how the use of each screen was largely mediated by the nurse's position on the left side of the bed near the patient chart, or on the right side of the bed near the vitals monitor. As described in Section 5.3, we see support for this analysis in the conditional gaze distribution, with fixations on the vitals screen going from 2.8 to 18.5% and 10.5 to 20%, when moving from left to right of the bed in scenarios 1 and 2, respectively.

However, based on this initial DiCoT analysis, we would also expect fixations on the patient chart to have the opposite relationship, decreasing significantly when moving from left to right of the bed. However, in our case studies, the fixations on the patient chart only significantly decreased in S1, moving from 25% on the left to 1.9% on the right, while in S2 the fixations on the patient chart decreased very slightly, with 22.8% on the left and 20% on the right. While it is clear that physical layout mediates the use of these information hubs, the data also suggests an additional latent mediating factor is present. We hypothesize that differences in the simulation scenarios contributed to this, with S2 requiring more references to the patient chart than S1, probably because of the incorrect diagnostic hypothesis the nurse initially made, but there are also other potential explanations, such as differences in the strategies adopted by the two nurses.

This is a simple example of a new insight generated by the quantitative methods that can lead to additional research to refine the qualitative models, but it also demonstrates the overall idea of the cyclic model design. After using the system to analyze learner data, we gain new insights that can be given back to simulation designers and researchers to help formulate new research questions and supporting simulation studies. We can iteratively update our qualitative understanding of simulation based on learner data, leading to better analysis of the data, and subsequent learner feedback, in the future.

## 7. Conclusions

In this paper, we presented an analysis of a nurse simulation-based training environment using multimodal learning analytics, cognitive task analysis, and distributed cognition analysis using the DiCoT framework. We show how the analysis of multimodal data from both qualitative and quantitative perspectives can be combined into a common framework for analyzing mixed-reality simulation-based training environments, such as the nursing case study analyzed here. While this work is still in its initial stages, the analysis methods developed and demonstrated here suggest a great potential for combining qualitative distributed cognition analysis with multimodal quantitative analytics in order to generate a more complete understanding of SBT as a whole. The strengths of each method are amplified when used together, and such an integrated approach can help shed new lights on simulation-based training and generate new insights.

However, this work and the framework it presents are not without limitations, and future work is required to address these concerns. One of the major limitations of the presented framework is its lack of guidance on the selection of adequate data sources and design of the associated analysis techniques. Since relevant data sources and analysis techniques differ widely among SBT domains, it is difficult to create a universal guidance on selection and design of these concepts while also keeping the domain-generality of the presented framework. In addition, this study was also limited by the sample size, only analyzing a small case-study of two simulation. This small study size allowed us to focus carefully on the design of the framework and the specific feature of the analysis, but limits the argument for the generalizability of the framework and the analysis results.

Future work will expand our study, both to more data from the nurse training simulation domain, as well as to a variety of other training domains. This expanded work will help to mitigate both of these limitations, as it will allow us to further validate the analysis methods across a wide variety of participants, as well as reveal commonalities among disparate training domains that can be used to generate guiding principles for the selection of adequate data sources and design of the associated analysis techniques. In addition, these further studies will place an emphasis on capturing data related to collaborative and teamwork activities in these environments, helping to further develop the distributed cognition frameworks that ground our data analysis techniques.

To support these expanded studies, future work will also focus on replacing the manual annotation of data used in this study with automated AI and machine learning techniques. Specifically, manual annotation was used in this study for the action, speech, and gaze modalities. For actions, techniques from video activity/action recognition will be applied to automatically extract time segments where the nurse is performing relevant actions (Ghadiyaram et al., 2019; Zhu et al., 2020). For speech, tagging will be automated using pre-trained natural language processing models, such as deep transformer models like Google BERT (Devlin et al., 2018), which have been fine-tuned on our specific domain. In addition, these pre-trained language models will also be applied toward a variety of other downstream NLP tasks, such as event detection and discourse analysis. For gaze, computer vision techniques will be used to automatically match the egocentric video to annotated static camera viewpoints, allowing us automatically determine specific objects (AOIs) that the nurse is looking at (Bettadapura et al., 2015).

Finally, this study and its associated framework was limited in guiding the design of formative learner feedback mechanisms based on the analysis. While Section 6 discussed some of the implications of the framework and its analysis on learning and pedagogy, including the possibility of developing formative learner feedback to support discussion sessions using contrasting cases, the framework itself does not detail guidance for designing learner feedback mechanisms. In addition, for this study specifically, analysis of the case-study data was performed *post-hoc*, so feedback based on the analysis could not be generated in-time for students. Future work, will automate the analysis methods, develop learning analytics to evaluate learner behaviors and actions, and will focus on presenting learners with online feedback designed to support simulation debriefing and after-action reviews. By presenting the results of our analysis to learners and instructors, we can get valuable feedback about the usability of the system and what types of feedback mechanisms might be relevant and important for these stakeholders to see in future iterations of the system.

## Data availability statement

The raw data supporting the conclusions of this article will be made available by the authors, without undue reservation.

## Ethics statement

The studies involving human participants were reviewed and approved by Vanderbilt University Institutional Review Board. The patients/participants provided their written informed consent to participate in this study. Written informed consent was obtained from the individual(s) for the publication of any potentially identifiable images or data included in this article.

## Author contributions

GB is the principle investigator of the study and contributed to its initial conceptualization and development of the analysis framework. CV, ED, and CC were responsible for data collection, annotation, and curation. NM maintained the computational and data infrastructure. CV performed the primary data analysis and wrote the initial draft of the manuscript. All authors contributed to model development, interpretation of results, manuscript revision, and approved the submitted version.

## Funding

This work represents independent research supported in part by Army Research Laboratory Award W912CG2220001 and NSF Cyberlearning Award 2017000, as well as equipment and funding from the Vanderbilt LIVE initiative.

## Conflict of interest

The authors declare that the research was conducted in the absence of any commercial or financial relationships that could be construed as a potential conflict of interest.

## Publisher's note

All claims expressed in this article are solely those of the authors and do not necessarily represent those of their affiliated organizations, or those of the publisher, the editors and the reviewers. Any product that may be evaluated in this article, or claim that may be made by its manufacturer, is not guaranteed or endorsed by the publisher.

## Author disclaimer

The views expressed in this paper do not necessarily reflect the position or policy of the United States Government or the National Science Foundation, and no official endorsement should be inferred.

## References

[B1] Al-Ghareeb A. Z. Cooper S. J. (2016). Barriers and enablers to the use of high-fidelity patient simulation manikins in nurse education: an integrative review. Nurse Educ. Today 36, 281–286. 10.1016/j.nedt.2015.08.005 26323885

[B2] Bettadapura V. Essa I. Pantofaru C. (2015). Egocentric field-of-view localization using first-person point-of-view devices, in 2015 IEEE Winter Conference on Applications of Computer Vision (Waikoloa, HI: IEEE), 626–633.

[B3] Biswas G. Rajendran R. Mohammed N. Goldberg B. S. Sottilare R. A. Brawner K.. (2019). Multilevel learner modeling in training environments for complex decision making. IEEE Trans. Learn. Technol. 13, 172–185. 10.1109/TLT.2019.2923352

[B4] Blandford A. Furniss D. (2006). Dicot: a methodology for applying distributed cognition to the design of teamworking systems, in Interactive Systems. Design, Specification, and Verification, eds Gilroy S. W. Harrison M. D. (Berlin; Heidelberg: Springer Berlin Heidelberg), 26–38.

[B5] Blikstein P. (2013). Multimodal learning analytics, in Proceedings of the Third International Conference on Learning Analytics and Knowledge (Leuven), 102–106.

[B6] Blikstein P. Worsley M. (2016). Multimodal learning analytics and education data mining: using computational technologies to measure complex learning tasks. J. Learn. Anal. 3, 220–238. 10.18608/jla.2016.32.11

[B7] Bransford J. D. Schwartz D. L. (1999). Chapter 3: rethinking transfer: a simple proposal with multiple implications. Rev. Res. Educ. 24, 61–100. 10.3102/0091732X024001061

[B8] Bylinskii Z. Borkin M. A. Kim N. W. Pfister H. Oliva A. (2015). Eye fixation metrics for large scale evaluation and comparison of information visualizations, in Workshop on Eye Tracking and Visualization (Cham: Springer), 235–255.

[B9] Clark A. (1997). Being There. Cambridge, MA: MIT Press.

[B10] Clark R. E. Estes F. (1996). Cognitive task analysis for training. Int. J. Educ. Res. 25, 403–417. 10.1016/S0883-0355(97)81235-9

[B11] Cochran K. Cohn C. Hutchins N. Biswas G. Hastings P. (2022). Improving automated evaluation of formative assessments with text data augmentation, in AIED Durham, NC.

[B12] Cole M. (1998). Cultural Psychology: A Once and Future Discipline. Cambridge; Massachusetts, MA; London: Harvard University Press.

[B13] Cook D. A. Zendejas B. Hamstra S. J. Hatala R. Brydges R. (2014). What counts as validity evidence? examples and prevalence in a systematic review of simulation-based assessment. Adv. Health Sci. Educ. 19, 233–250. 10.1007/s10459-013-9458-4 23636643

[B14] CooperJ.TaquetiV. (2008). A brief history of the development of mannequin simulators for clinical education and training. Postgrad Med. J. 84, 563–570. 10.1136/qshc.2004.00988619103813

[B15] Daniels K. Auguste T. (2013). Moving forward in patient safety: multidisciplinary team training. Semin. Perinatol. 37, 146–50. 10.1053/j.semperi.2013.02.004 23721769

[B16] Devlin J. Chang M.-W. Lee K. Toutanova K. (2018). BERT: pre-training of deep bidirectional transformers for language understanding. arXiv e-prints, arXiv:1810.04805. 10.48550/arXiv.1810.0480535689168

[B17] Di Mitri D. Schneider J. Specht M. Drachsler H. (2019). Detecting mistakes in cpr training with multimodal data and neural networks. Sensors 19, 3099. 10.3390/s19143099 31337029 PMC6679577

[B18] Feinstein A. H. Cannon H. M. (2002). Constructs of simulation evaluation. Simulat. Gaming 33, 425–440. 10.1177/1046878102238606

[B19] Fraser K. L. Ayres P. Sweller J. (2015). Cognitive load theory for the design of medical simulations. Simulat. Healthcare 10, 295–307. 10.1097/SIH.0000000000000097 26154251

[B20] Freedman D. H. (2010). Why scientific studies are so often wrong: the streetlight effect. Discover Mag. 26, 1–4. Available online at: https://www.discovermagazine.com/the-sciences/why-scientific-studies-are-so-often-wrong-the-streetlight-effect

[B21] Fu H. Wu L. Jian M. Yang Y. Wang X. (2019). Mf-sort: simple online and realtime tracking with motion features, in International Conference on Image and Graphics (Springer: Beijing), 157–168.

[B22] Galliers J. Wilson S. Fone J. (2007). A method for determining information flow breakdown in clinical systems. Int. J. Med. Inform. 76, S113-S121. 10.1016/j.ijmedinf.2006.05.015 16815738

[B23] Geertz C. (1973). The growth of culture and the evolution of mind, in The Interpretation of Cultures (New York, NY), 76.

[B24] Gegenfurtner A. Quesada-Pallarès C. Knogler M. (2014). Digital simulation-based training: a meta-analysis. Br. J. Educ. Technol. 45, 1097–1114. 10.1111/bjet.12188

[B25] Ghadiyaram D. Tran D. Mahajan D. (2019). Large-scale weakly-supervised pre-training for video action recognition, in Proceedings of the IEEE/CVF Conference on Computer Vision and Pattern Recognition (Long Beach, CA), 12046–12055.

[B26] Hazlehurst B. Gorman P. N. McMullen C. K. (2008). Distributed cognition: an alternative model of cognition for medical informatics. Int. J. Med. Inform. 77, 226–234. 10.1016/j.ijmedinf.2007.04.008 17556014

[B27] Hegland P. A. Aarlie H. Strømme H. Jamtvedt G. (2017). Simulation-based training for nurses: systematic review and meta-analysis. Nurse Educ. Today 54, 6–20. 10.1016/j.nedt.2017.04.004 28456053

[B28] Hollan J. Hutchins E. Kirsh D. (2000). Distributed cognition: toward a new foundation for human-computer interaction research. ACM Trans. Comput. Hum. Interact. 7, 174–196. 10.1145/353485.353487

[B29] Hoppe H. U. (2017). Computational methods for the analysis of learning and knowledge building communities, in Handbook of Learning Analytics (Beaumont, AB), 23–33.

[B30] Hutchins E. (1991). The social organization of distributed cognition, in Perspectives on Socially Shared Cognition (Washington, DC: American Psychological Association), 283–307.

[B31] Hutchins E. (1995). Cognition in the Wild. Cambridge, MA: MIT Press.

[B32] Hutchins E. (2000). Distributed cognition, in International Encyclopedia of the Social and Behavioral Sciences (Amsterdam: Elsevier Science), 138.

[B33] Hutchins E. (2006). The distributed cognition perspective on human interaction. Roots Hum. Soc. 1, 375. 10.4324/9781003135517-19

[B34] Johnson M. P. Hickey K. T. Scopa-Goldman J. Andrews T. Boerem P. Covec M.. (2014). Manikin versus web-based simulation for advanced practice nursing students. Clin. Simulat. Nurs. 10, e317-e323. 10.1016/j.ecns.2014.02.004

[B35] Jørnø R. L. Gynther K. (2018). What constitutes an ‘actionable insight' in learning analytics? J. Learn. Anal. 5, 198–221. 10.18608/jla.2018.53.13

[B36] Kang S. J. Min H. Y. (2019). Psychological safety in nursing simulation. Nurse Educ. 44, E6-E9. 10.1097/NNE.0000000000000571 30052586

[B37] Kaplan A. D. Cruit J. Endsley M. Beers S. M. Sawyer B. D. Hancock P. (2021). The effects of virtual reality, augmented reality, and mixed reality as training enhancement methods: a meta-analysis. Hum. Factors 63, 706–726. 10.1177/0018720820904229 32091937

[B38] Kim J. W. Sottilare R. A. Brawner K. Flowers T. (2018). Integrating sensors and exploiting sensor data with gift for improved learning analytics, in Sixth Annual GIFT Users Symposyum Orlando, FL.

[B39] Kunst E. L. Mitchell M. Johnston A. N. (2016). Manikin simulation in mental health nursing education: an integrative review. Clin. Simulat. Nurs. 12, 484–495. 10.1016/j.ecns.2016.07.010

[B40] Laerdal Medical (2022a). LLEAP - Laerdal Learning Application Stavanger: Laerdal Medical.

[B41] Laerdal Medical (2022b). SimMan 3G Advanced Patient Simulator Stavanger: Laerdal Medical.

[B42] Liu B. Zhao Q.-C. Ren Y.-Y. Wang Q.-J. Zheng X.-L. (2018). An elaborate algorithm for automatic processing of eye movement data and identifying fixations in eye-tracking experiments. Adv. Mech. Eng. 10, 1687814018773678. 10.1177/1687814018773678

[B43] López M. X. Strada F. Bottino A. Fabricatore C. (2021). Using multimodal learning analytics to explore collaboration in a sustainability co-located tabletop game, in European Conference on Games Based Learning (Brighton: Academic Conferences International Limited), 482-XXI.

[B44] Maran N. J. Glavin R. J. (2003). Low-to high-fidelity simulation-a continuum of medical education? Med. Educ. 37, 22–28. 10.1046/j.1365-2923.37.s1.9.x 14641635

[B45] Martinez-Maldonado R. Echeverria V. Fernandez Nieto G. Buckingham Shum S. (2020a). From data to insights: a layered storytelling approach for multimodal learning analytics, in Proceedings of the 2020 Chi Conference on Human Factors in Computing Systems (Honolulu, HI), 1–15.

[B46] Martinez-Maldonado R. Elliott D. Axisa C. Power T. Echeverria V. Buckingham Shum S. (2020b). Designing translucent learning analytics with teachers: an elicitation process, in Interactive Learning Environments, 1–15. 10.1080/10494820.2019.1710541

[B47] Meakim C. Boese T. Decker S. Franklin A. Gloe D. Lioce L.. (2013). Standards of best practice: simulation standard i: Terminology. Clin. Simulat. Nurs. 9, S3-S11. 10.1016/j.ecns.2013.04.00126958174

[B48] Militello L. G. Hutton R. J. (1998). Applied cognitive task analysis (acta): a practitioner's toolkit for understanding cognitive task demands. Ergonomics 41, 1618–1641. 10.1080/001401398186108 9819578

[B49] Mirchi N. Bissonnette V. Yilmaz R. Ledwos N. Winkler-Schwartz A. Del Maestro R. F. (2020). The virtual operative assistant: an explainable artificial intelligence tool for simulation-based training in surgery and medicine. PLoS ONE 15, e0229596. 10.1371/journal.pone.0229596 32106247 PMC7046231

[B50] Ochoa X. Lang A. C. Siemens G. (2017). Multimodal learning analytics. Handbook Learn. Anal. 1, 129–141. 10.18608/hla17.011

[B51] Olsen A. (2012). The tobii ivt fixation filter algorithm. Los Altos, CA: Technical report, Tobii Pro.

[B52] Otter.ai (2022). Otter.ai-Voice Meeting Notes and Real-time Transcription. Los Altos, CA.

[B53] Pardo A. (2018). A feedback model for data-rich learning experiences. Assess. Evaluat. Higher Educ. 43, 428–438. 10.1080/02602938.2017.1356905

[B54] Park J. E. Kim J.-H. (2021). Nursing students experiences of psychological safety in simulation education: a qualitative study. Nurse Educ. Pract. 55, 103163. 10.1016/j.nepr.2021.103163 34333233

[B55] Pimmer C. Pachler N. Genewein U. (2013). Reframing clinical workplace learning using the theory of distributed cognition. Acad. Med. 88, 1239–1245. 10.1097/ACM.0b013e31829eec0a 23887014

[B56] Ravert P. (2002). An integrative review of computer-based simulation in the education process. Comput. Inform. Nurs. 20, 203–208. 10.1097/00024665-200209000-00013 12352106

[B57] Rokhsaritalemi S. Sadeghi-Niaraki A. Choi S.-M. (2020). A review on mixed reality: current trends, challenges and prospects. Appl. Sci. 10, 636. 10.3390/app10020636

[B58] Rosen M. A. Salas E. Wilson K. A. King H. B. Salisbury M. Augenstein J. S.. (2008). Measuring team performance in simulation-based training: adopting best practices for healthcare. Simulat. Healthcare 3, 33–41. 10.1097/SIH.0b013e3181626276 19088640

[B59] Rybing J. (2018). Studying Simulations With Distributed Cognition, Vol. 1913. Linköping: Linköping University Electronic Press.

[B60] Rybing J. Nilsson H. Jonson C.-O. Bang M. (2016). Studying distributed cognition of simulation-based team training with dicot. Ergonomics 59, 423–434. 10.1080/00140139.2015.1074290 26275026

[B61] Rybing J. Prytz E. Hornwall J. Nilsson H. Jonson C.-O. Bang M. (2017). Designing a digital medical management training simulator using distributed cognition theory. Simulat. Gaming 48, 131–152. 10.1177/1046878116676511

[B62] Sawyer T. L. Deering S. (2013). Adaptation of the us army's after-action review for simulation debriefing in healthcare. Simulat. Healthcare 8, 388–397. 10.1097/SIH.0b013e31829ac85c 24096913

[B63] Schraagen J. M. Chipman S. F. Shalin V. L. (2000). Cognitive Task Analysis. New York, NY: Psychology Press.

[B64] Stanton N. A. (2014). Representing distributed cognition in complex systems: how a submarine returns to periscope depth. Ergonomics 57, 403–418. 10.1080/00140139.2013.772244 23510256

[B65] Stenetorp P. Pyysalo S. Topić G. Ohta T. Ananiadou S. Tsujii J. (2012). brat: a web-based tool for NLP-assisted text annotation, in Proceedings of the Demonstrations Session at EACL 2012 (Avignon: Association for Computational Linguistics), 102–107.

[B66] Sun Z. Chen J. Chao L. Ruan W. Mukherjee M. (2020). A survey of multiple pedestrian tracking based on tracking-by-detection framework. IEEE Trans. Circ. Syst. Video Technol. 31, 1819–1833. 10.1109/TCSVT.2020.3009717

[B67] Tobii Pro (2012). Determining the Tobii I-VT Fixation Filter's Default Values. Danderyd Municipality: Tobii Technology

[B68] Tobii Pro (2022). Tobii Pro Glasses 3. Danderyd Municipality: Tobii Technology.

[B69] Vatral C. Biswas G. Goldberg B. S. (2022). Multimodal learning analytics using hierarchical models for analyzing team performance, in Proceedings of the 15th International Conference on Computer Supported Collaborative Learning (Hiroshima: International Society of the Learning Sciences).

[B70] Vatral C. Mohammed N. Biswas G. Goldberg B. S. (2021). Gift external assessment engine for analyzing individual and team performance for dismounted battle drills, in Proceedings of the 9th Annual Generalized Intelligent Framework for Tutoring User Symposium (Orlando, FL: US Army Combat Capabilities Command Center), 109–127.

[B71] Wojke N. Bewley A. Paulus D. (2017). Simple online and realtime tracking with a deep association metric, in 2017 IEEE International Conference on Image Processing (ICIP) (Beijing: IEEE), 3645–3649.

[B72] Worsley M. Martinez-Maldonado R. (2018). Multimodal learning analytics' past, present, and potential futures, in CrossMMLA@ LAK (Sydney), 1–16.

[B73] Wright P. C. Fields R. E. Harrison M. D. (2000). Analyzing human-computer interaction as distributed cognition: the resources model. Hum. Comput. Interact. 15, 1–41. 10.1207/S15327051HCI1501_0114552843

[B74] Zachary W. W. Ryder J. M. Hicinbothom J. H. (2000). Building cognitive task analyses and models of a decision-making team in a complex real-time environment, in Cognitive Task Analysis (New York, NY), 365–384.

[B75] Zhu Y. Li X. Liu C. Zolfaghari M. Xiong Y. Wu C.. (2020). A comprehensive study of deep video action recognition. arXiv preprint 1–30. 10.48550/arXiv.2012.06567

